# Candidate SNP Markers of Atherogenesis Significantly Shifting the Affinity of TATA-Binding Protein for Human Gene Promoters Show Stabilizing Natural Selection as a Sum of Neutral Drift Accelerating Atherogenesis and Directional Natural Selection Slowing It

**DOI:** 10.3390/ijms21031045

**Published:** 2020-02-05

**Authors:** Mikhail Ponomarenko, Dmitry Rasskazov, Irina Chadaeva, Ekaterina Sharypova, Irina Drachkova, Dmitry Oshchepkov, Petr Ponomarenko, Ludmila Savinkova, Evgeniya Oshchepkova, Maria Nazarenko, Nikolay Kolchanov

**Affiliations:** 1 Institute of Cytology and Genetics, Siberian Branch of Russian Academy of Sciences, Novosibirsk 630090, Russia; rassk@bionet.nsc.ru (D.R.); ichadaeva@bionet.nsc.ru (I.C.); sharypova@bionet.nsc.ru (E.S.); drachkova@bionet.nsc.ru (I.D.); diman@bionet.nsc.ru (D.O.); pon_petr@mail.ru (P.P.); lksav@bionet.nsc.ru (L.S.); nzhenia@bionet.nsc.ru (E.O.);; 2 Novosibirsk State University, Novosibirsk 630090, Russia; 3 Institute of Medical Genetics, Tomsk National Research Medical Center, Russian Academy of Science, Tomsk 634009, Russia; maria.nazarenko@medgenetics.ru

**Keywords:** atherosclerosis, human, gene, promoter, TATA-binding protein (TBP) TBP-binding site (TATA box), single nucleotide polymorphism (SNP), candidate SNP marker, verification in vitro

## Abstract

(1) Background: The World Health Organization (WHO) regards atherosclerosis-related myocardial infarction and stroke as the main causes of death in humans. Susceptibility to atherogenesis-associated diseases is caused by single-nucleotide polymorphisms (SNPs). (2) Methods: Using our previously developed public web-service SNP_TATA_Comparator, we estimated statistical significance of the SNP-caused alterations in TATA-binding protein (TBP) binding affinity for 70 bp proximal promoter regions of the human genes clinically associated with diseases syntonic or dystonic with atherogenesis. Additionally, we did the same for several genes related to the maintenance of mitochondrial genome integrity, according to present-day active research aimed at retarding atherogenesis. (3) Results: In dbSNP, we found 1186 SNPs altering such affinity to the same extent as clinical SNP markers do (as estimated). Particularly, clinical SNP marker rs2276109 can prevent autoimmune diseases via reduced TBP affinity for the human *MMP12* gene promoter and therefore macrophage elastase deficiency, which is a well-known physiological marker of accelerated atherogenesis that could be retarded nutritionally using dairy fermented by lactobacilli. (4) Conclusions: Our results uncovered SNPs near clinical SNP markers as the basis of neutral drift accelerating atherogenesis and SNPs of genes encoding proteins related to mitochondrial genome integrity and microRNA genes associated with instability of the atherosclerotic plaque as a basis of directional natural selection slowing atherogenesis. Their sum may be stabilizing the natural selection that sets the normal level of atherogenesis.

## 1. Introduction

Atherosclerosis is an inflammatory disorder of arteries that can lead to myocardial infarction and stroke (i.e., two most frequent causes of death in humans according to the World Health Organization (WHO) [1]). The current conventional view is that low-density lipoprotein accumulation near the vessel inner wall is the precondition for atherogenesis initiation [2]. Next, monocytes surround such lipoprotein clusters to absorb them and differentiate into macrophage foam cells, which can separate and take away excess lipoproteins temporarily as well as return them with lipoproteins deficit for their homeostasis and so on until such cells die over time, thereby building these clusters up into fatty streaks [3]. Incidentally, such streaks can gradually transform first into thrombogenic bands, then into their fibrous agglomerates, and finally, into atherosclerotic plaques, which can be calcified and become inflammation hotbeds within blood vessels [4]. Eventually, this process leads to thrombosis of the artery, and as a result, to the death of the affected tissue [5], in particular, myocardial infarction or stroke [1]. Clinical observations indicate that atherogenesis is a nonmonotonic slow step-by-step cyclic process developing throughout the lifespan mainly postprandially (i.e., after a meal [6]) during acute infectious events [7] near an injury site in the vessel endothelium during hypertension [2,4]. Indeed, precursors of fatty streaks in an embryo have been clinically documented [8], whereas atherosclerosis is mostly a disease of old age [1]. Thus, at any given moment, the current atherogenic status of an individual reflects all his/her living conditions, lifestyle, and diseases in previous years, which can occasionally accelerate or retard atherogenesis depending on his/her atherogenesis susceptibility in accordance with his/her individual genome [2]. Therefore, the sequenced genome of an individual allows to increase his/her life expectancy via the slowing of his/her atherogenesis if this person chooses the living conditions and lifestyle that can minimize dietary fat, infectious diseases, extreme physical exertion, injuries, foreign substances in the blood, and the risks of comorbidities related to atherosclerosis.

This is what predictive-preventive personalized participatory (4P) medicine can already do [9]. Its keystone is the top scientific project of the 21st century: “1000 Genomes” [10]; under this project, scientists have already sequenced thousands of individual genomes and assembled them into a reference human genome (i.e., Ensembl [11]) and variome (i.e., dbSNP [12] containing hundreds of millions of single-nucleotide polymorphisms [SNPs]) and made them publicly available via the UCSC Genome Browser [13]. Additionally, to help physicians to deal with individual genomes of their patients, scientists working on database dbWGFP [14] continuously search for, compile, systematize, and prioritize any available information on each of 10^10^ potential SNPs throughout the human genome, as do the researchers behind two databases: ClinVar [15] and OMIM [16], containing only clinically documented and only well-studied SNPs, respectively.

According to OMIM data [16], the absolute majority of well-studied SNPs are within protein-coding regions of human genes, where they damage protein structure and/or functions without fail throughout the human body. These SNPs seem easy to detect but cannot be neutralized either therapeutically or by changing living conditions or the lifestyle (see, e.g., in [17]) without repair of the damage to gene sequences; this field is only beginning to be studied on laboratory animals and is widely discussed hypothetically in relation to humans. On the contrary, a negligible percentage of well-studied SNPs is within regulatory gene regions [16,18], where, without damaging a protein, they can modulate only gene expression levels, which vary from cell to cell, from tissue to tissue, and so on for many reasons; these SNPs are very hard to detect but easy to neutralize by medications, living conditions, and the lifestyle. Indeed, exogenous insulin successfully helps those with hypoinsulinemia who have no insulin resistance and who try to lead a lifestyle that does not provoke acute aggravation of this disorder. Of note, the best-studied regulatory SNPs are often located within a 70 bp proximal core promoter [19], where they modulate both TATA-binding protein (TBP) binding affinity for these promoters and transcription activity of these promoters (these parameters are proportionally related [20]), thereby acting as indispensable switches between inactive nucleosome packages and active preinitiation complexes [21].

The key concept of 4P medicine [9] is a clinical SNP marker of a given disease; alleles of this SNP allow to statistically significantly distinguish between representative cohorts of patients with this disease and conventionally healthy volunteers as the only acceptable criterion; this is because it quantifies the expected likelihood of a medical error associated with each clinical SNP marker used (see, e.g., in [22]). Nonetheless, it takes too much time, manual labor, and money to test how each of 10^10^ human SNPs [14] affects each of 55,000 diseases (see ICD-11 [23]), and why should we if Kimura’s theory [24] and Haldane’s dilemma [25] predict no phenotypic manifestations for the vast majority of such SNPs.

As a first step, why not try cheap and fast bioinformatic genome-wide calculations to find this vast majority of neutral SNPs in humans? This approach should exclude them from clinical studies, which consequently could become more targeted and less expensive. Although the accuracy of current genome-wide computational predictions remains below the applicability threshold for clinical SNP tests [26], this accuracy increases every year [27,28,29,30,31,32].

In our previous studies, we first measured the TBP-promoter affinity in vitro [33,34,35], next revealed partial correlations “sequence => activity” in silico [34], and then generalized them as the three-step TBP-promoter binding process (i.e., TBP slides along DNA <=> TBP stops at a TBP-binding site (TBP-site) <=> the TBP-promoter complex is fixed by a 90° bend of DNA [36]) as observed in vitro [37]. After that, we nevertheless verified our three-step model using all 68 independent experimental datasets that we could find within the PubMed database [38] (for review, see [39]) as well as using our own experiments under equilibrium [40], nonequilibrium [41], and real-time conditions [42,43,44] in vitro. On this basis, we created our publicly available Web service SNP_TATA_Comparator [45] whose input data are two DNA sequences, namely, one for the ancestral allele of the promoter under study, and the other for the minor allele of this promoter. Using these input data, SNP_TATA_Comparator calculates TBP binding affinity estimates for two corresponding promoter variants and standard errors of these estimates as well as statistical significance of the difference between them according to the Fisher Z-test [45]. Next, we validated the selected predictions of SNP_TATA_Comparator [45] in our own experiments ex vivo by means of human cell lines transfected with the pGL4.10 vector carrying a reporter *LUC* gene under the control of a promoter containing the SNP in question (for review, see [46]). In this way, we have repeatedly confirmed that the increase in the TBP-promoter affinity estimate predicted by our SNP_TATA_Comparator [45] statistically significantly corresponds to an increase in the expression level of the reporter gene regulated by this promoter and vice versa [46].

Finally, we applied this software to predict candidate SNP markers of resistance to anticancer therapy [47], obesity [48], and autoimmune [49] and Alzheimer’s [50] diseases as well as markers of nonclinical aberrations in humans such as circadian rhythm disturbances [51], aggressiveness [52], female reproductive potential anomalies [53], and domination and subordination within a social hierarchy [54]. In these works of ours, we studied only SNPs located within 70 bp proximal promoter regions in front of transcription start sites of the human genes because TBP-sites are necessary here [21]. Among such TBP-binding promoter regions, the most valuable for us are those that contain known clinically proven SNP markers of a human pathology that reliably change TBP-binding affinity for the corresponding promoters and expression levels of the human genes regulated by these promoters in accordance with the clinically confirmed markers of these pathologies. This information allows us to most reliably predict clinical manifestations of other SNPs in such a promoter region that alter TBP-binding affinity for this promoter as do clinically proven SNP markers located within this promoter (according to our calculations using SNP_TATA_Comparator [45]). Therefore, we constantly update our collection of such clinically proven SNP markers of human pathologies on the basis of our curated search for relevant articles in the PubMed database [38] as well as other publicly available databases (for example, ClinVar [15]), and we have published its updates in many studies depending on the human pathologies considered there.

Our recent study on this topic is about 34 candidate SNP markers of atherosclerosis [55] that are located around the clinical SNP markers in TBP-sites of 17 human gene promoters. These clinical SNP markers seem to be subject to neutral drift as genetic loads, according to the statistical estimates from our previous works [49,50,51,54] within the framework of Kimura’s theory [24] and Haldane’s dilemma [25] using a previous dbSNP build (No. 147 dated 2016) [12].

Here, we followed the same approach by means of current dbSNP build No. 151 dated 2017 [12] (i.e., almost four times the size of the previous build, No. 147) to compare our results updated in this way with our above-mentioned previous results [55]. This approach allowed us to examine the robustness of genome-wide patterns [49,50,51,54] of the candidate SNP markers predicted by our SNP_TATA_Comparator [45] in the face of annual growth of the SNP count.

Finally, we analyzed several genes encoding proteins related to mitochondrial genome integrity [56] and microRNA (miRNA, miR) genes associated with instability of the atherosclerotic plaque [57] that are currently widely studied regarding the slowing of atherogenesis.

## 2. Results and Discussion

To verify the robustness of our Web service predictions in terms of the annual SNP count growth, using current dbSNP build No. 151 [12], we analyzed 644 SNPs within 70 bp proximal promoters of 26 human genes, where clinical SNP markers are located according to our previous reviews of this topic [19,21,45], which are now updated here (i.e., *ACKR1*, *ADH7*, *APOA1*, *CETP*, *COMT*, *DHFR*, *ESR2*, *F3*, *F7*, *FGFR2*, *GCG*, *HBB*, *HBD*, *HSD17B1*, *HTR2C*, *IL1B*, *INS*, *LEP*, *MBL2*, *MLH1*, *MMP12*, *NOS2*, *RET*, *SOD1*, *TGFBR2*, and *TPI1*), and we compared the results with those from build No. 147 [12]. The present analysis yielded 145 candidate SNP markers for atherosclerosis among 644 SNPs examined (23%) versus 34 candidate markers among 175 SNPs (19%) in our previous work [55], as readers can see in in rows 3 and 4 of Table 1.

In addition, with the help of our SNP_TATA_Comparator [45], we analyzed all the 464 and 81 SNPs in question within all known promoters of four protein-coding genes and four miRNA genes, respectively, which comprehensively exemplify two gene networks related to the maintenance of mitochondrial genome integrity [56] and instability of the atherosclerotic plaque [57] in humans. As a result, we predicted 77 and 16 candidate SNP markers of this disorder and characterized them (rows 5 and 6 of Table 1).

We will describe in depth only our predictions regarding human gene *CETP*, whose promoter contains the only clinically proven SNP marker of atherosclerosis, so that we next review all our other predictions briefly in a similar way.

Finally, we will evaluate them all as a whole regarding their statistical significance with respect to the widely accepted genome-wide patterns of SNP occurrence.

### 2.1. The Only Clinically Proven SNP Marker of Atherosclerosis in a TBP-Site of a Human Gene Promoter

**Figure 1:**
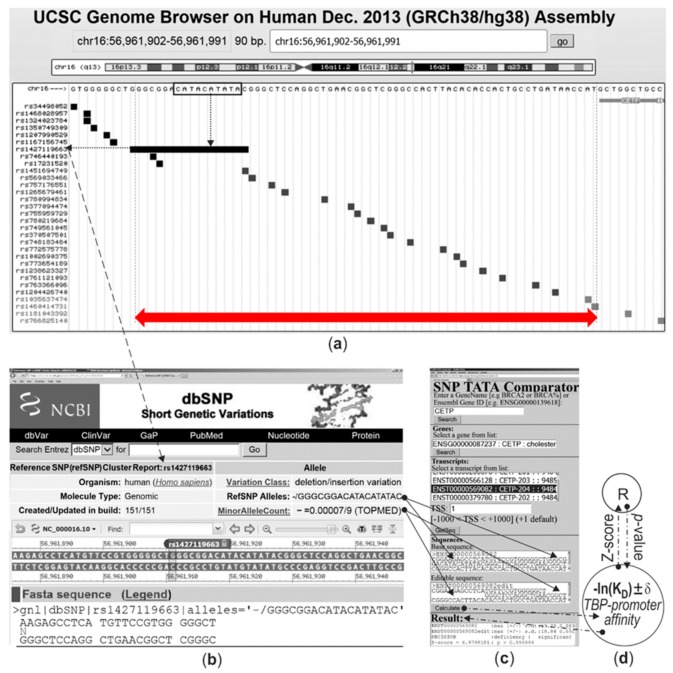
The result produced by our SNP_TATA_Comparator [45] in the case of the only known clinical SNP marker (rs1427119663) of slowed atherosclerosis in the human *CETP* gene. Legend: (**a**) Unannotated SNPs (analyzed in this study) in a 70 bp proximal promoter [where all proven TBP-sites (framed, □) are located (double-headed red arrow, ↔) of the human *CETP* gene retrieved from the UCSC Genome Browser [13]. (**b**) The description of this clinical SNP marker (rs1427119663) of retarded atherosclerosis corresponds to dbSNP build No. 151 [12]. (**c**) The output of our public Web service SNP_TATA_Comparator [45] after the input of data on the clinical SNP marker rs1427119663 as depicted by arrows (i.e., the textbox Result: “deficiency: significant” in line “Decision”). (d) Our software-based calculation implementing our bioinformatics model of the three-step TBP-promoter binding (i.e., TBP slides along DNA <=> TBP stops at a TBP-site <=> the TBP-promoter complex is fixed by a 90° DNA bend [36]), based on standard bioinformatics-related software R [59], as described in detail within Supplementary Materials (Supplementary File S1: Supplementary Methods).

Human gene *CETP* (plasma lipid transfer protein) carries the only known clinical SNP marker rs1427119663 (i.e., 18 bp deletion 5′-G_71_GGCGGACATACATATAC_54–_3′ containing the TBP-site within the 70 bp proximal promoter region of this gene: a frame (□) and a double-headed red arrow, respectively, in Figure 1a), which reduces CETP expression and thus retards atherogenesis [58], as depicted by the “↓” symbol (down arrow) in Table 2. Figure 1 illustrates how we predicted this rs1427119663-dependent CETP underexpression, when we retrieved the proper input data from (a) the UCSC Genome Browser [13] via (b) the dbSNP database [12] and entered these data into (c) our SNP_TATA_Comparator [45], which processed them using (d) standard bioinformatics-related software R [59], as indicated by the arrows there. This correct in silico prediction (Figure 1) of our SNP_TATA_Comparator [45] in the case of the only known clinical SNP marker of atherosclerosis (Table 2) indicates its applicability to atherogenesis research.

Near the known SNP marker (rs1427119663), there are 22 SNPs (Figure 1a), none of which have been characterized so far regarding any link to human diseases (for brevity, here we will refer to such SNPs as unannotated). Among these 22 unannotated SNPs, using SNP_TATA_Comparator [45], we predicted four SNPs (e.g., rs1002690375) that can cause *CETP* overexpression as a physiological marker of accelerated atherogenesis [60]. Accordingly, we can suggest four candidate SNP markers of accelerated atherogenesis, which are italicized in Table 2. To tell the reader about the effect of each predicted candidate SNP marker on gene expression in comparison either to the clinical SNP marker used or to one another, we prioritized these predictions heuristically using the *p*-value of Fisher’s Z-test in terms of heuristic rank ρ-values, which vary in alphabetical order from “A” (the best) to “E” (the worst; Table 2: column ρ). Within our example considered, we designated the known clinically proven SNP marker rs1427119663 as “the best”, whereas another SNP (candidate marker rs569033466) received a lower rating, “B”.

Finally, using a PubMed keyword search [38], we found that swimming can slow atherogenesis down [61].

**Table 1:** Atherogenesis-related candidate SNP markers within the gene promoters, where SNPs of TBP-sites are clinically linked with the human diseases; their comparison with the genome-wide patterns.

Data: GRCh38, dbSNP Rel. 151 [12]				Result	H_0_: Neutral Drift [24,25]			H_0_: ↑ and ↓ Equivalence		
*n*	SNPs	N_GENE_	N_SNP_	N_RES_	*n*_>_	*n*_<_	*P*(H_0_: *n*_>_ < *n*_<_) [62]	*n*_↑_	*n*_↓_	*P*(H_0_: *n*_↑_ ≡ *n*_↓_)
1	Whole-genome norm for SNPs of TBP-sites [63]	10^4^	10^5^	10^3^	200	800	>0.99	-	-	-
2	Clinically known SNP markers of diseases at TBP-sites [45]	33	203	51	14	37	>0.99	-	-	-
3	Candidate SNP markers for atherogenesis near clinical SNP markers for diseases at TBP-sites [55]	17	175	34	7	27	>0.99	19	15	>0.30
4	Candidate SNP markers for atherogenesis near clinical SNP markers for diseases at TBP-sites [this work]	26	644	145	72	73	0.50	91	54	<0.01
5	Candidate SNP markers for atherogenesis near the TBP-sites of protein-coding genes related to the maintenance of mitochondrial genome integrity [56], this work	4	464	77	48	29	<0.05	27	50	<0.01
6	Candidate SNP markers of atherogenesis near the TBP-sites of the miRNA genes associated with instability of atherosclerotic plaque [57], this work	4	81	16	11	5	<0.05	2	14	<0.01
7	TOTAL	34	1189	238	-	-	-	-	-	-

Notes. Atherogenesis: accelerated (↑) and slowed down (↓), N_GENE_ and N_SNP_: total numbers of the human genes and of their SNPs meeting the criteria of this study. N_RES_: the total number of the candidate SNP markers predicted in this work that can increase (*n*_>_) or decrease (*n*_<_) the affinity of TATA-binding protein (TBP) for these promoters and to, respectively, affect the expression of these genes. *n*_↑_ and *n*_↓_: the total numbers of the candidate SNP markers that can accelerate or slow down atherogenesis, respectively. P(H_0_), the estimate of probability for the acceptance of this H_0_ hypothesis, according to a binomial distribution; TBP-site, TATA-binding protein-binding site.

**Table 2:** Atherogenesis-related candidate SNP markers within the gene promoters where SNPs of TBP-sites are clinically linked with cardiovascular diseases.

*Gene*	dbSNP ID [12] or [Ref]	DNA, Genome Sequence				K_D_, nM						Clinical Data or *Candidate SNP Markers*	AS	Ref.
		5′ flank	WT	min	3′ flank	WT	min	Δ	Z	α	ρ			
*CETP*	rs1427119663	cgtgggggct	18bp	-	gggctccagg	4	7	<	7	10^−6^	A	hyperalpha-lipoprotei-nemia (athero-protector)	*↓*	[58]
	*rs1002690375*	*gggccactta*	*c*	*t*	*acaccactgc*	*4*	*2*	*> *	*11*	*10^−6^*	*A*	*accelerated atherogenesis, which can be slowed down by swimming*	*↑*	[61,62]
	*rs757176551*	*catatacggg*	*c*	*g*	*tccaggctga*	*4*	*2*	*> *	*10*	*10^−6^*	*A*		*↑*	
	*rs569033466*	*atacatatac*	*g*	*a, t, c*	*ggctccaggc*	*4*	*3*	*> *	*4*	*10^−3^*	*B*		*↑*	
	*rs1451694749*	*catacatata*	*c*	*t*	*gggctccagg*	*4*	*2*	*> *	*12*	*10^−6^*	*A*		*↑*	
*MBL2*	rs72661131	tctatttcta	t	c	tatagcctgc	2	4	<	12	10^−6^	A	stroke, pre-eclampsia, variable immune-deficiency	*↑*	[64,65,66]
	*rs1471733364*	*tatttctata*	*t*	*g*	*agcctgcacc*	*2*	*5*	*< *	*15*	*10^−6^*	*A*	*on the Western diet, MBL2 deficit and excess quicken earlier and late atherogenesis, respectively*	*↑*	[67,68,69]
	*rs562962093*	*atctatttct*	*a*	*g*	*tatagcctgc*	*2*	*5*	*< *	*15*	*10^−6^*	*A*		*↑*	
	*rs567653539*	*tttctatata*	*g*	*a*	*cctgcaccca*	*2*	*1*	*> *	*4*	*10^−3^*	*B*		*↑*	
*F3*	rs563763767	ccctttatag	c	t *	gcgcggggca	3	2	>	6	10^−6^	A	myocardial infarction, thrombosis	*↑*	[70]
	*rs1439518731*	*gccctttata*	*-*	*ta*	*gcgcgcgggg*	*3*	*1*	*> *	*12*	*10^−6^*	*A*	*accelerated atherogenesis that can be slowed down by aspirin* *slowed down atherogenesis*	*↑*	[71,72]
	*rs966076891:t*	*gccgccggcc*	*c*	*t*	*tttatagcgc*	*3*	*2*	*> *	*6*	*10^−6^*	*A*		*↑*	
	*rs966076891:g*	*gccgccggcc*	*c*	*g*	*tttatagcgc*	*3*	*4*	*< *	*2*	*0.05*	*D*		*↓*	[73]
	*rs1190659847*	*gccggccctt*	*t*	*c*	*atagcgcgcg*	*3*	*12*	*< *	*19*	*10^−6^*	*A*		*↓*	
*TPI1*	rs1800202	gcgctctata	t	g	aagtgggcag	1	4	<	17	10^−6^	A	hemolytic anemia, neu-romuscular diseases	*↑*	[74,75]
	*rs1386262216*	*gcggcgctct*	*a*	*c, g*	*tataagtggg*	*1*	*2*	*< *	*8*	*10^−6^*	*A*	*traumatic and ischemic co-mplications of atherogenesis*	*↑*	[76,77]
	*rs781835924*	*cgcggcgctc*	*t*	*c*	*atataagtgg*	*1*	*2*	*< *	*10*	*10^−6^*	*A*		*↑*	

Notes. Hereinafter, Alleles: wt, ancestral; min, minor; “-”, deletion. K_D_, dissociation constant of the TBP–DNA complex; α = 1 – p, significance (where p value is given in Figure 1); Gene expression changes (Δ): an increase (>) and decrease (<); Atherogenesis (AS): accelerated (↑) and slowed down (↓); ρ, heuristic rank of candidate SNP markers from the “best” (A) to the “worst” (E). * This SNP also includes other neutral alleles. Diseases: RA, rheumatoid arthritis. Genes: *CETP*, cholesteryl ester transfer protein; *MBL2*, mannose-binding lectin 2 (synonyms: collectin-1); *F3*, coagulation factor III (synonyms: thromboplastin, tissue factor); *TPI1*, triosephosphate isomerase 1. Deletions, *CETP*: 18bp = gggcggacatacatatac.

### 2.2. Human Genes Associated with Cardiovascular Diseases

Human gene *MBL2* encodes mannose-binding lectin 2 and has a clinically proven SNP marker (rs72661131) of stroke [64], preeclampsia [65], and variable immunodeficiency [66] due to MBL2 deficiency, as shown in Table 2. By searching PubMed [38], we found a retrospective clinical review [67] on MBL2 insufficiency as a physiological marker of atherogenesis speeding up via both formation and destabilization of atherosclerotic plaques in the course of thrombogenesis. With this in mind and without any cause–effect assumptions, we predicted that this known clinical SNP marker rs72661131 can also be a candidate SNP marker of acceleration of late atherogenesis (Table 2).

Around a known SNP marker, rs72661131, we found an unannotated SNP (rs567653539) causing MBL2 overexpression as well as two unannotated SNPs (rs1471733364 and rs562962093) causing its underexpression (Table 2). Using PubMed search software [38], we learned that the MBL2 excess and deficit correspond to acceleration of early and late atherogenesis in rheumatoid arthritis [68] and on a Western-pattern (standard American) diet [69]. That is why we predicted three more candidate SNP markers (rs567653539, rs1471733364, and rs562962093) of increased atherogenesis (Table 2).

Human gene *F3* (thromboplastin) contains clinically proven SNP marker rs563763767 increasing the expression of this gene thus causing either myocardial infarction or thrombosis [70] as complications of atherogenesis [71], which can be prevented by low-dose aspirin therapy [72], according to searches in PubMed [38]. Near rs563763767, there are two unannotated SNPs—rs1439518731 and rs966076891:t—that can also increase the thromboplastin level and thus cause these diseases as atherogenesis complications (Table 2). In addition, within the same promoter, we found two more unannotated SNPs (rs966076891:g and rs1190659847) able to reduce thromboplastin expression, whereas antithromboplastin therapy slows down atherogenesis in a murine model of human atherosclerosis [73], as we learned in the PubMed database [38] (Table 2). Summing up all our findings about the human *F3* gene within this table, we predicted five candidate SNP markers of atherogenesis types listed there.

Human gene *TPI1* coding for triosephosphate isomerase includes a known SNP marker (rs1800202) reducing this enzyme’s amount [74], and thereby leading to hemolytic anemia and neuromuscular diseases [75], which correspond to traumatic complications of atherogenesis [76] and a risk factor for atherosclerosis development [77], according to PubMed [38]. In the vicinity of rs1800202, we uncovered two unannotated SNPs (rs1386262216 and rs781835924) that can lead to TPI1 underexpression too. This finding allows us to propose them as candidate SNP markers of enhanced atherogenesis for the same reasons [74,75,76,77], as one can see in Table 2.

### 2.3. Human Genes Associated with Blood Disorders

Human genes *HBB* and *HBD* correspond to β- and δ-subunits of hemoglobin, promoters of which carry the greatest number (seven) of clinical SNP markers (e.g., *HBB*: rs33981098 and *HBD*: rs35518301) of hemoglobin insufficiency responsible for malaria resistance and thalassemia [78], which are atheroprotective [79]. Therefore, they can all be candidate SNP markers of delayed atherogenesis (Table 3). Looking through these promoters, we found four unannotated SNPs (e.g., *HBB*: rs281864525 and *HBD*: rs996092254), which can also cause hemoglobin deficiency, and therefore may be candidate SNP markers of an atherogenesis slowdown (Table 3).

**Figure 2:**
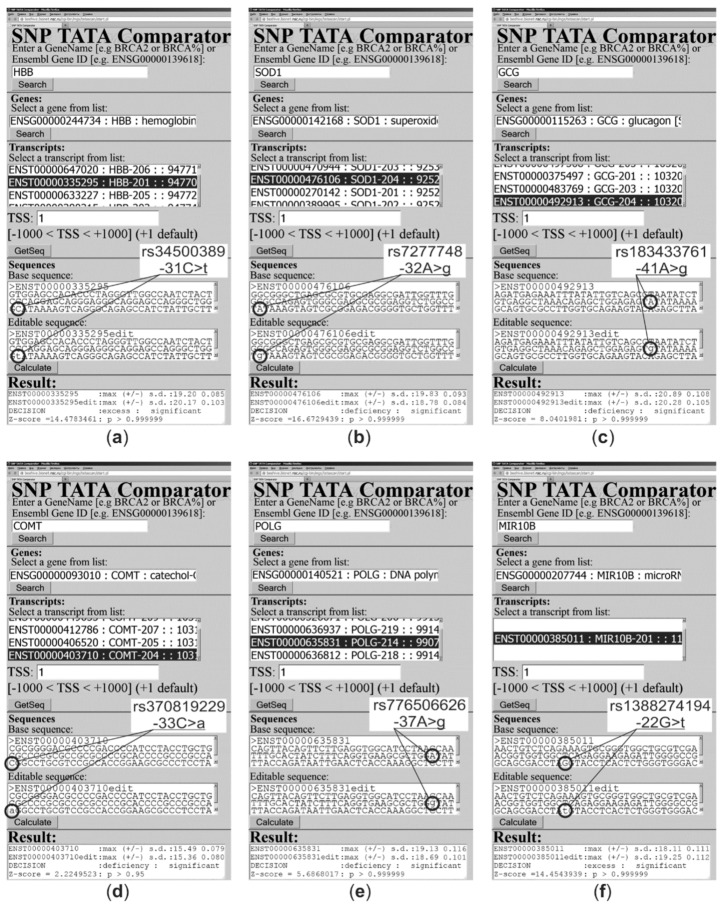
Examples of predictions made in this work (using our previously created Web service SNP_TATA_Comparator [45]) regarding statistically significant changes in the expression of human genes. *Legend*: See the footnote of Figure 1. Candidate SNP markers of accelerated and retarded atherogenesis: (**a**) *HBB*: rs34500389: −31C>T; (**b**) *SOD1*: rs7277748: −32A>G; (**c**) GCG: rs183433761: −41A>G; (**d**) *COMT*: rs370819229: −33C>A; (**e**) *POLG*: rs776506626: −37A>G; (**f**) *MIR10B*: rs1388274194: −22G>T.

In addition, there is substitution A>T at position −27 of the *HBB* promoter (hereinafter: *HBB*:−27A>T) [80] (not covered by either the “1000 Genomes” project [10] or dbSNP [12]), which causes *HBB* overexpression that has been clinically associated with the norm of the two above-mentioned biomedical traits [80] (Table 3). Nonetheless, our keyword search via PubMed [38] led to a clinical research article [81] on the heme released by extracellular HBB (after hemolysis), as an accelerator of atherogenesis. That is why we predicted that the known SNP marker HBB:−27A>T of the norm of both malaria resistance and thalassemia [80] can also be a candidate SNP marker of accelerated atherogenesis (Table 3). Finally, within promoters in question, we found three more unannotated SNPs (e.g., HBB: rs34500389, our prediction for which is presented in Figure 2a, as an illustrative example) that can raise the expression of the genes in question too. On this basis, we proposed three candidate SNP markers of accelerated atherogenesis, which are listed in Table 3.

**Table 3:** Atherogenesis-related candidate SNP markers within the gene promoters where SNPs of TBP-sites are clinically linked with blood disorders.

*Gene*	dbSNP ID [12] or [Ref]	DNA, Genome Sequence				K_D_, nM						Clinical Data or *Prediction*	AS	Ref.
		5′ flank	WT	min	3′ flank	WT	min	Δ	Z	α	ρ			
*HBB*	rs33981098	agggctgggc	a	g, c	taaaagtcag	5	9	<	10	10^−6^	A	thalassemia, RM	↓	[78]
	rs33980857	gggctgggca	t	a, t, g	aaaagtcagt	5	21	<	27	10^−6^	A		↓	
	rs397509430	gggctgggca	t	-	aaaagtcagt	5	29	<	34	10^−6^	A		↓	
	rs34598529	ggctgggcat	a	g	aaagtcaggg	5	18	<	24	10^−6^	A		↓	
	rs33931746	gctgggcata	a	g, c	aagtcagggc	5	11	<	14	10^−6^	A		↓	
	*rs281864525*	*tgggcataaa*	*a*	c *	*gtcagggcag*	*5*	*7*	*< *	*7*	*10^−6^*	*A*	*thalassemia is athero-protective*	↓	[79]
	*rs63750953*	*ctgggcataa*	*aa*	*-*	*gtcagggcag*	*5*	*8*	*< *	*9*	*10^−6^*	*A*		↓	
	*rs1160543272:t*	*ccagggctgg*	*g*	*t*	*cataaaagtc*	*4.6*	*5.3*	*< *	*2*	*0.05*	*D*		↓	
	*rs1160543272:a*	*ccagggctgg*	*g*	*a*	*cataaaagtc*	*4.6*	*3.8*	*> *	*3*	*10^−2^*	*C*	norm (silent SNP), *hemo-lytically extra-cellular HBB releases athe-rogenic heme*	↑	[80,81]
	*rs34500389*	*cagggctggg*	*c*	t^*^	*ataaaagtca*	*5*	*2*	*> *	*14*	*10^−6^*	*A*		↑	
	[80]	gctgggcata	a	t	aagtcagggc	5	3	>	8	10^−6^	A		↑	
*HBD*	*rs996092254*	*aggacaggac*	*c*	*t*	*agcataaaag*	4	3	*> *	*4*	*10^−6^*	*A*		↑	
	*rs1473693473*	*ataaaaggca*	*a*	*g*	*ggcagagtcg*	4	5	*< *	*3*	*10^−2^*	*C*	*thalassemia athero-protective*	*↓*	[79]
	*rs34166473*	*aggaccagca*	*t*	*c*	*aaaaggcagg*	4	12	*< *	*18*	*10^−6^*	*A*		*↓*	
	rs35518301	caggaccagc	a	g	taaaaggcag	4	8	<	11	10^−6^	A	thalassemia, RM	↓	[78]
*ACKR1*	rs2814778	ttggctctta	t	c	cttggaagca	10	12	<	4	10^−3^	B	leukopenia, RM	↓	[82,83]
												*slowed down atherogenesis*		[84]
	*rs1185314734*	*tggctcttat*	*c*	*g*	*ttggaagcac*	*10*	*6*	*> *	*8*	*10^−6^*	*A*	*accelerated atherogenesis*	↑	[85]
*NOS2*	[30]	gtataaatac	t	c	tcttggctgc	1.7	1.4	>	3	10^−2^	C	RM	↑	[86,87]
												*accelerated atherogenesis*		[88,89]
	*rs1339255364*	*gcatggggtg*	*a*	g *	*gtataaatac*	*1.7*	*2.0*	*< *	*2*	*0.05*	*D*	*slowed down atherogenesis*	↓	[90]
*F7*	[91]	ccttggaggc	a	c	gagaactttg	53	62	<	3	10^−2^	C	moderate bleeding	↑	[91]
												*atherogenesis slowed by dill*		[92,93]
	*rs749691733*	*agaactttgc*	*c*	*t*	*cgtcagtccc*	*53*	*66*	*< *	*4*	*10^−3^*	*B*	*F7 excess is a biomarker of hypercholes-terolemia, which accelerates atherogenesis, which can be slowed down by olive oil *	↑	[94,95]
	*rs997515289*	*ttggaggcag*	*a*	*c*	*gaactttgcc*	*53*	*68*	*< *	*5*	*10^−3^*	*B*		↑	
	*rs549591993*	*gcccgtcagt*	*c*	*a*	*ccatggggaa*	*53*	*25*	*> *	*13*	*10^−6^*	*A*		↑	
	*rs367732974*	*aactttgccc*	*g*	*a*	*tcagtcccat*	*53*	*47*	*> *	*2*	*0.05*	*D*		↑	
	*rs777947114*	*agagaacttt*	*g*	*a*	*cccgtcagtc*	*53*	*19*	*> *	*19*	*10^−6^*	*A*		↑	
	*rs1187329967*	*tggaggcaga*	*g*	*c*	*aactttgccc*	*53*	*32*	*> *	*9*	*10^−6^*	*A*		↑	
	*rs770113559*	*gtcacccttg*	*g*	*a*	*aggcagagaa*	*53*	*41*	*> *	*5*	*10^−6^*	*A*		↑	
	*rs781338265*	*cctctgtcac*	*c*	a *	*cttggaggca*	*53*	*30*	*> *	*11*	*10^−6^*	*A*		↑	
	*rs754814507*	*cctcccccat*	*c*	*t*	*cctctgtcac*	*53*	*45*	*> *	*3*	*10^−3^*	*B*		↑	
	*rs1296764751*	*tcccctcccc*	*c*	*t*	*atccctctgt*	*53*	*46*	*> *	*2*	*0.05*	*A*		↑	

Diseases: RM, resistance to malaria. Genes: *HBB* and *HBD*, hemoglobin subunits β and δ; *ACKR1*, atypical chemokine receptor; *NOS2*, nitric oxide synthase 2; *F7*, coagulation factor VII.

Human gene *ACKR1* (atypical chemokine receptor 1, synonyms: glycoprotein D, Duffy blood group) contains the known SNP marker (rs2814778) of leukopenia [82] and resistance to malaria [83] due to low TBP-promoter affinity, and therefore ACKR1 underexpression. Thanks to PubMed [38], we learned that ACKR1 underexpression reduces atherogenesis in a mouse model of human atherogenesis [84]. For this reason, we suggested rs2814778 as a candidate SNP marker of an atherogenesis slowdown (Table 3). By analyzing the same promoter, we selected an unannotated SNP, rs1185314734, corresponding to glycoprotein D overexpression, which is a known physiological marker of atherosclerosis-related cardiovascular diseases, according to a retrospective clinical review [85] that was found using the PubMed keyword search utility [38] (Table 3).

The promoter of human gene *NOS2* carries a known SNP marker (NOS2:−51T>C) of malaria resistance [86,87], which is caused by overexpression of inducible nitric oxide synthase encoded by this gene. Due to PubMed [38], we know that an NO excess is an accelerator of atherogenesis according to both physiological [88] and nutritional [89] original research articles. Therefore, the known SNP marker (NOS2:−51T>C) of malaria resistance [86,87] could be additionally regarded as a candidate SNP marker of accelerated atherogenesis (Table 3).

Additionally, looking through the same promoter, we encountered an unannotated SNP, rs1339255364, which can reduce NOS2 expression, whereas any inhibition of NOS2 yields an atherosclerosis slowdown in line with a mouse model of human atherosclerosis [90], as revealed by a keyword search in PubMed [38]. With this in mind, we propose that rs1339255364 is a candidate SNP marker of retarded atherogenesis as readers can see in Table 3.

Human gene *F7* (coagulation factor VII, synonym: serum prothrombin conversion accelerator) contains a clinically proven SNP marker (F7:−33A>C) of moderate bleeding [91] owing to F7 underexpression (Table 3). Using a keyword search in PubMed [38], we unexpectedly found clinical data on both F7 deficit [92] and F7 excess [93] as atherogenic factors, whose negative consequences are preventable by two atheroprotective diets recommend by nutritionists [94,95]. Consequently, we predicted 11 candidate SNP markers of accelerated atherogenesis within the *F7* promoter under study, namely, the above-mentioned substitution F7:−33A>C, two unannotated SNPs reducing the F7 level in the blood (e.g., rs749691733), and eight unannotated SNPs elevating it (e.g., rs549591993, Table 3).

### 2.4. Human Genes Associated with Autoimmunity-Associated Diseases

Human gene *SOD1* encodes Cu/Zn superoxide dismutase and carries a well-known SNP marker (rs7277748) of this gene’s underexpression (Figure 2b) and of familial amyotrophic lateral sclerosis [96] as a risk factor of atherosclerosis development; this complication is often provoked by various autoimmune diseases according to epidemiological reviews [97,98,99] (Table 4). Near rs7277748, there are three unannotated SNPs (e.g., rs1438766715 as a 26 bp deletion of the wild type TBP-site of this promoter), which can reduce the SOD1 level and thus have the same clinical manifestations (Table 4). Thus, we suggest them all as candidate SNP markers of atherogenesis acceleration. Finally, due to PubMed keyword search software [38], we know that short-term physical exercise [100], onion extract nutritional supplements [101], and antioxidants [102] can slow down these health problems (Table 4).

Human gene *INS* (insulin) contains a known SNP marker (rs5505) of neonatal diabetes mellitus mediated by hyperinsulinemia [15], which in turn is a well-known physiological marker of atherosclerosis [103,104]. Around rs5505, we selected three more unannotated SNPs able to cause the same hyperinsulinemia (e.g., rs1389349459) and thereby atherogenesis (Table 4). In addition, here we revealed the only unannotated SNP (rs11557611) associated with hypoinsulinemia, and this SNP speeds up atherogenesis too [105]. As readers can see in Table 4, within the examined promoter region of this gene, we identified all five candidate SNP markers of accelerated atherogenesis only. Nevertheless, thanks to the PubMed keyword search utility [38], we eventually learned about a dietary soy isoflavone as a norminsulinemic atheroprotector [106].

**Table 4:** Atherogenesis-related candidate SNP markers within the gene promoters where SNPs of TBP-sites are clinically linked with autoimmunity-associated diseases.

Gene	dbSNP ID [12] or [Ref]	DNA, Genome Sequence				K_D_, nM						Clinical Data or Candidate SNP Markers	AS	Ref.
		5′ flank	WT	min	3′ flank	WT	min	Δ	Z	α	ρ			
*SOD1*	rs7277748	ggtctggcct	a	g	taaagtagtc	2	7	<	17	10^−6^	A	familial amyotrophic lateral sclerosis	↑	[96,97,98,99]
	*rs1438766715*	*agagtgggcg*	*26bp*	*-*	*agtcgcggag*	*2*	*79*	*<*	*59*	*10^−6^*	*A*	*accelerated atherogenesis that can be slowed down by short-term exercise or onion extract*	↑	[100,101,102]
	*rs1325052558*	*tctggcctat*	*a*	*g*	*aagtagtcgc*	*2*	*9*	*<*	*22*	*10^−6^*	*A*		↑	
	*rs966452334*	*aggtctggcc*	*t*	*c*	*ataaagtagt*	*2*	*6*	*<*	*16*	*10^−6^*	*A*		↑	
*INS*	rs5505	agatcactgt	c	t	cttctgccat	53	44	>	4	10^−3^	B	neonatal diabetes mellitus, hyper(pro)-insulinemia	↑	[15]
	*rs1389349459*	*ctgtctccca*	*g*	*t*	*atcactgtcc*	*53*	*14*	*>*	*25*	*10^−6^*	*A*	*atherogenesis that can be slowed by dietary soy isoflavone as norminsuli-nemic athero-protector*	↑	[103,104,106]
	*rs563207167*	*tcagccctgc*	*c*	*t*	*tgtctcccag*	*53*	*44*	*>*	*4*	*10^−3^*	*B*		↑	
	*rs1367101897*	*cctcagccct*	*g*	*a*	*cctgtctccc*	*53*	*32*	*>*	*9*	*10^−6^*	*A*		↑	
	*rs11557611*	*gatcactgtc*	*c*	*t*	*ttctgccatg*	*53*	*60*	*<*	*2*	*0.05*	*D*		↑	[105]
*MMP12*	rs2276109	gatatcaact	a	g	tgagtcactc	11	14	<	3	10^−2^	C	lower risks of psoriasis, systemic scleroderma, and asthma	↑	[107,108,109,110]
	*rs572527200*	*gatgatatca*	*a*	*g*	*ctatgagtca*	*11*	*14*	*<*	*3*	*10^−2^*	*C*	*accelerated atherogenesis that can be slowed by milk fermented with lacto-bacilli and dietary sup-plements akin to estrogens*	↑	[111,112]
	*rs1401366377*	*tatcaactat*	*g*	*a*	*agtcactcat*	*11*	*3*	*>*	*20*	*10^−6^*	*A*		↑	[113,114]

Genes: *SOD1*, superoxide dismutase 1; *INS*, insulin; *MMP12*, matrix metallopeptidase 12 (synonym: macrophage elastase). Deletions, *SOD1*: 26bp = aggcgcggaggtctggcctataaagt.

Human gene *MMP12* contains a clinical SNP marker (rs2276109) of lower risk of many immune diseases, such as psoriasis [107], systemic scleroderma [108], and asthma [109], because of underexpression of the macrophage elastase encoded by this gene. Because MMP12 performs degradation of fibrinogen in the blood [110,111], its underexpression accelerates atherogenesis. Within the same promoter, we identified one more unannotated SNP (rs572527200) reducing MMP12 abundance, which can also be a candidate SNP marker of atherogenesis acceleration (Table 4). In addition, here we found one more unannotated SNP (rs1401366377) elevating the macrophage elastase level, which is known as a physiological marker of accelerated progression of atherosclerosis in transgenic rabbits [112] (Table 4). Looking through our predictions about this gene, we see all three candidate SNP markers accelerating atherogenesis only, whereas the PubMed keyword search utility [38] pointed us to milk products fermented by lactobacilli [113] and estrogen-like nutritional supplements [114], which can decelerate these health problems (Table 4).

### 2.5. Human Genes Associated with Obesity

Human gene *GCG* encodes glucagon, a so-called hunger hormone, because feelings of hunger decrease with a decrease in its concentration [115], i.e., during hypoglucagonemia [116] (Table 5), which can be caused by any of the five unannotated SNPs that we found in the promoter of this gene (e.g., rs183433761, as shown in Figure 2c). After searching PubMed, we learned that atherogenesis is accelerated postprandially in proportion to the ratio of insulin concentration to glucagon concentration [117]. This observation allows us to propose five candidate SNP markers of accelerated atherogenesis (Table 5).

As for our keyword search in PubMed [38], in this way, we found a sport medicine article on some atherosclerosis-related postprandial effects, namely, in childhood, high-fat food ingestion just before physical exercise can slow down both growth and development [118].

Human gene *LEP* codes for leptin, which is also called obesity hormone because laboratory animals with a protein-damaging mutation in this gene are abnormally obese [119], as shown in Table 5. As readers can see in this table, within the human leptin promoter, we identified two unannotated SNPs able to cause obesity that can accelerate atherogenesis in line with a review article [120] found in PubMed using its keyword search utility [38]. In addition, here we found three unannotated SNPs increasing the LEP level (and thus protecting against both obesity and atherosclerosis [121]) as candidate SNP markers of an atherogenesis slowdown (e.g., rs34104384, Table 5).

Human gene *APOA1* for apolipoprotein A1 carries a known SNP marker (APOA1:−35A>C) of hematuria, fatty liver, and obesity [122], and is a commonly accepted risk factor of atherogenesis acceleration [120]. Indeed, this prediction is consistent with the outcome of treatment of atherosclerosis using exogenous apolipoprotein A1 [123] as well as by both a low-protein diet and exercise increasing the endogenous APOA1 level in obese post-menopausal women [124]. Near this substitution APOA1:−35A>C, we found three more unannotated SNPs, which can cause APOA1 underexpression and, as a consequence, obesity and atherosclerosis. Therefore, we propose them as candidate SNP markers of accelerated atherogenesis (e.g., rs1428975217), as readers can see in Table 5.

Human gene *HTR2C* (5-hydroxytryptamine receptor 2C, synonym: serotonin receptor 2C) contains a known SNP marker (rs3813929) of overexpression of this gene causing obesity as a complication of antipsychotic treatment with olanzapine [15]. This SNP can therefore speed up atherogenesis, consistently with the above-mentioned review [120] and independent clinical data [125,126] (Table 5). Around rs3813929, we recognized five unannotated SNPs (e.g., rs1348095721), all of which seem to yield HTR2C overexpression too, thereby allowing us to recommend them as candidate SNP markers of an atherogenesis increase, as presented in Table 5.

Human gene *IL1B* encodes interleukin 1β and has a known biomedical SNP marker (rs1143627) of IL1B overexpression causing the greatest number and variety of human diseases, including obesity [127], Graves’ disease [128], major recurrent depression [129], non-small cell lung cancer [130], hepatocellular carcinoma in hepatitis C virus infection [131], gastric cancer [132], gastric ulcer, and chronic gastritis [133] (Table 5). Using the known association between this clinical SNP marker and above-mentioned obesity and independent single-cell RNA-seq data on IL1B excess as a pro-atherogenic factor [134], we can recommend verifying it as another candidate SNP marker of atherogenesis acceleration (Table 5).

Within the *IL1B* promoter region in question, we found the only unannotated SNP (rs549858786), which can reduce the expression of this gene (Table 5). As for our keyword search in PubMed [38], it resulted in a pharmaceutical original research article [135] on IL1B deficiency as a physiological marker of successful treatment of atherosclerosis using oligomeric proanthocyanidins from *Rhodiola rosea*, as detailed in Table 5. This time, we surprisingly found an article [136] on proinflammatory effects of cigarette smoke condensates as an atherogenic risk factor, suggesting that passive smoking can be as dangerous as active one.

### 2.6. Human Genes Associated with Carcinogenesis

Human gene *ADH7* (alcohol dehydrogenase 7) contains a clinically proven SNP marker (rs17537595) of ADH7 underexpression responsible for esophageal cancer [137], as shown in Table 6. According to the outcome of our keyword search in PubMed [38], post-esophagectomy necrosis is comorbid with atherosclerosis in this case [138]. This result allows us to propose this known SNP marker of carcinogenesis as a candidate SNP marker of accelerated atherogenesis (Table 6).

Finally, within the *ADH7* gene promoter, we found three unannotated SNPs, which can also reduce its expression and thus speed up atherogenesis too (e.g., rs372329931, Table 6).

Human gene *HSD17B1* codes for hydroxysteroid (17-β) dehydrogenase 1, whose deficit in the case of the well-known SNP marker rs201739205 is clinically detectable in patients with hereditary breast cancer [139], as readers can see in Table 6. Due to a PubMed search, we learned about insufficiency of this enzyme as a physiological marker of a successful antiatherosclerotic therapy based on the IMM-H007 drug [140]. That is why we predicted a candidate SNP marker (rs201739205) of an atherogenesis slowdown (Table 6). Furthermore, near it, we found two unannotated SNPs, which can also reduce this enzyme’s level and therefore may be candidate SNP markers of atherogenesis retardation (Table 6; e.g., rs748743528).

Finally, within the *HSD17B1* promoter region in question, we detected three unannotated SNPs able to raise the HSD17B1 level (e.g., rs1282820277), which is a well-known physiological marker of accelerated atherogenesis [141] according to PubMed [38] (Table 6). On this basis, we predicted three candidate SNP markers of atherogenesis acceleration due to an HSD17B1 excess (Table 6).

Human gene *MLH1* encodes DNA mismatch repair protein MLH1, and its promoter contains two clinically confirmed SNP markers (rs63750527 and rs756099600) of nonpolyposis colon cancer [15] because an excess of this repair protein can prevent cancer cell apoptosis during either an immune response or anticancer chemotherapy (Table 6), which is also known as a physiological marker of atherogenesis retardation [142,143]. With this in mind, we predicted two candidate SNP markers (rs63750527 and rs756099600) of atherogenesis deceleration (Table 6). Near them, we selected three unannotated SNPs, which seem to increase this reparatory protein’s abundance (e.g., rs753671152), and thus to retard atherosclerosis development as candidate SNP markers of an atherogenesis delay (Table 6). In addition, there we found three other unannotated SNPs, which decrease *MLH1* expression and, on the contrary, accelerate atherogenesis (e.g., rs587778905 as a 21 bp deletion with the wild type TBP-site and its local surroundings within the promoter being considered), which can be tested as candidate SNP markers for atherogenesis acceleration (Table 6). Finally, in this case, our keyword search in PubMed [38] unexpectedly returned a nutritional original research article [144] about atheroprotective effects of restrictions on alcohol drinking and red meat intake, according to their effects to DNA mismatch repair status.

Human gene *RET* has two clinically documented SNP markers—rs10900296 and rs10900297—corresponding to deficiency and excess of the Ret proto-oncogene encoded by this gene [15] (Table 6). Because of our keyword search in PubMed [38], we know about atheroprotective abilities of RET, whose deficiency dysregulates atheroprotector pentraxin-3 and vice versa [145]. With this in mind, we predicted a couple (rs551321384 and rs1191017949) of candidate SNP markers of atherogenesis acceleration during RET downregulation caused by them, whereas two others (rs1237152255 and rs1372293149) can be candidate SNP markers of slower atherogenesis due to a RET excess as their manifestation (Table 6).

Human gene *ESR2* (estrogen receptor 2 (β)) contains a clinical SNP marker (rs35036378) of an ESR2-deficient primary pT1 tumor of the mammary gland [146], whereas an ESR2 deficit is also a well-known physiological marker of the calcification stage of atherogenesis [147] (Table 6). Thus, it can be considered a candidate SNP marker of enhanced atherogenesis (Table 6). Around rs35036378, we chose the only unannotated SNP (rs766797386), which can reduce ESR2 concentration similarly to the above-mentioned candidate SNP marker of atherogenesis acceleration (Table 6). As for PubMed keyword search results [38], nutritionists recommend bioactivated calcium (Ca) in natural food as an atheroprotective nutrient in contrast to Ca-enriched dietary supplements elevating the risk of coronary artery calcification [148].

Human gene *DHFR* codes for dihydrofolate reductase and carries a known SNP marker (rs10168) of methotrexate resistance during anticancer chemotherapy because of this enzyme’s overexpression [149], which is in turn well known as an atheroprotector [150,151]. For this reason, we suggest a candidate SNP marker (rs10168) of retarded atherogenesis, as readers can see in Table 6. Near rs10168, we noticed the only unannotated SNP, rs750793297, which can also elevate the DHFR level, and thus be a candidate SNP marker of atherogenesis deceleration (Table 6). Finally, looking through this DHFR promoter, we recognized five unannotated SNPs reducing this enzyme’s abundance such as candidate SNP markers of atherogenesis acceleration (e.g., rs1464445339) that could be slowed down by restricting both active and secondhand smoking because they reduce the blood folate level [151] (Table 6).

### 2.7. Human Genes Associated with Developmental Disorders

Human gene *COMT* carries two known SNP markers (rs370819229 and rs777650793) corresponding to dilated cardiomyopathy and cardiovascular disease [15] as a consequence of either a deficit or excess of catechol-O-methyltransferase encoded by this gene (Table 7). Using PubMed [38], we learned that both a substrate (estradiol) and metabolite (2-methoxy estradiol) of this enzyme are atheroprotectors [152]. That is why we propose any SNP-caused statistically significant changes in the COMT level as candidate SNP markers of an atherogenesis delay (Table 7, e.g., rs370819229 exemplified in Figure 2d).

Human gene *TGFBR2* (transforming growth factor beta receptor 2) carries a clinical SNP marker (rs138010137) of underexpression of this gene resulting in aortic thoracic aneurysm and dissection [15]. Our keyword search in PubMed [38] resulted in an original research article on cellular genetics [153] about a TGFBR2 deficit provoking uncontrolled T-cell activation and maturation, which yields an inflammatory atherosclerotic plaque phenotype during hypercholesterolemia (Table 7). Consequently, we proposed s138010137 as a candidate SNP marker of accelerated atherogenesis (Table 7). Next, in the vicinity of rs138010137, we chose the only unannotated SNP rs1300366819, which can decrease TGFBR2 expression as a candidate SNP marker of atherogenesis acceleration (Table 7).

Finally, there we found another unannotated SNP, rs1310294304, which, on the contrary, can cause overexpression of this gene, as presented in Table 7. Nevertheless, the outcome of our keyword search in PubMed [38] revealed an association of TGFBR2 overexpression with hypertension, which is a well-known atherogenic risk factor [154]. Therefore, we predict that rs1310294304 is a candidate SNP marker of atherogenesis acceleration, as one can see in Table 7.

Human gene *FGFR2* encodes fibroblast growth factor receptor 2 (synonyms: keratinocyte growth factor receptor, bacteria-expressed kinase) and carries two clinically proven SNP markers, namely, rs886046768 for FGFR2 overexpression associated with craniosynostosis and rs777650793 for its downregulation linked to bent bone dysplasia [15] (Table 7). Thanks to the PubMed keyword search utility [38], we found an original experimental research article on a murine model of human atherosclerosis, where a synthetic small-molecule FGFR2 antagonist called SSR128129E successfully blocked atherogenesis [155], enabling us to propose rs886046768 and rs777650793 as candidate SNP markers of atherogenesis acceleration and retardation, respectively (Table 7).

Similarly, within promoters of this gene, we found six and three unannotated SNPs increasing and decreasing its expression, respectively, as candidate SNP markers of speeding up atherogenesis (e.g., rs1212347974) and an atherogenesis slowdown (e.g., rs1377663539), which are listed in Table 7. Finally, this sort of atherogenesis acceleration has been successfully retarded using a heparin-derived oligosaccharide in a rat model of the human atherosclerosis [156], as we found after a keyword search in PubMed [38].

### 2.8. Selective In Vitro Validation

**Figure 3:**
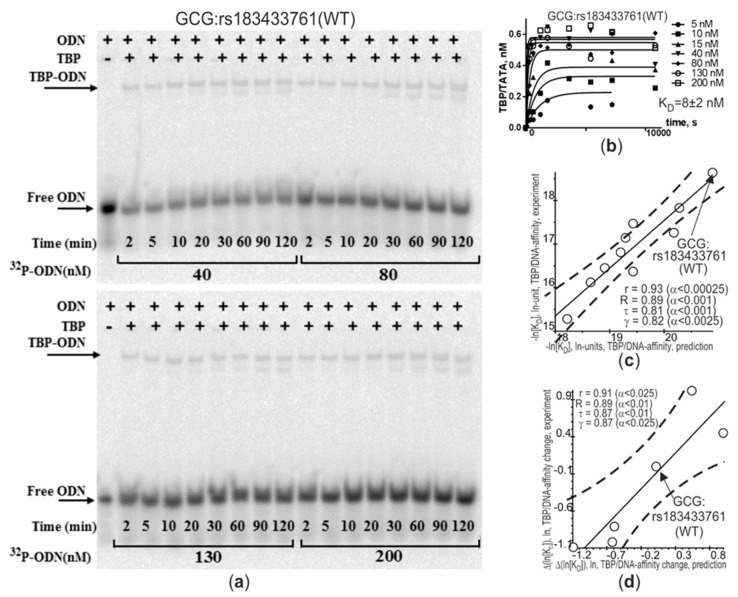
Statistically significant correlations between in silico predicted and in vitro measured values of equilibrium dissociation constant K_D_ of TBP-promoter complexes expressed in ln units. *Legend*: (**a**) Electropherograms for the wild type ancestral (WT) allele of the unannotated SNP rs183433761 in the human *GCG* gene promoter as an illustrative example. The concentration of TBP was 0.3 nM in all the experiments. The concentrations of 26 bp oligodeoxyribonucleotides (ODNs) centered around the tested SNP allele are indicated. (**b**) Dependences of reaction rates on ODN concentrations in cases of the ancestral alleles of the SNP in question. The KD value of the equilibrium dissociation constant was inferred from these dependences serving as input data for publicly available software GraphPad Prism 5 (http://graphpad-prism.software.informer.com/5.01). (**c**,**d**) Graphical representation of the analyzed correlations on absolute and relative measurement scales, respectively. Solid and dashed lines denote linear regression and boundaries of its 95% confidence interval, calculated by means of software package STATISTICA (Statsoft^TM^, Tulsa, USA). Arrows pinpoint the ancestral (WT) alleles of the SNP being studied (GCG:rs183433761). Statistics: r, τ, γ, and p are coefficients of Pearson’s linear correlation, Spearman’s rank correlation, Kendall’s rank correlation, and Goodman–Kruskal generalized correlation and their *p*-values, respectively.

After our bioinformatics prediction of 145 candidate SNP markers of atherogenesis near the clinically proven ones (Table 2, Table 3, Table 4, Table 5, Table 6 and Table 7), we first selectively verified them using an electrophoretic mobility shift assay (EMSA) under nonequilibrium conditions in vitro, as described in the section Materials and Methods. The primary experimental data in vitro on the five selected candidate SNP markers of atherogenesis predicted here—i.e., *GCG*: rs183433761, *LEP*: rs201381696, *HBB*: rs34500389, *HBD*: rs35518301, and *F3*: rs563763767—are exemplified by *GCG*: rs183433761 in Figure 3a,b. Finally, Figure 3c,d present their comparison with the predictions made in this work.

As readers can see in Figure 3c, according to five statistical criteria—i.e., Pearson’s linear correlation (r), the Goodman–Kruskal generalized correlation (γ), and Spearman’s (R) and Kendall’s (τ) rank correlations—our predictions are in significant agreement with the data measured experimentally in terms of absolute values of equilibrium dissociation constant K_D_ of TBP-promoter complexes, which are expressed in natural logarithm units. Independently, for the same five statistical tests, Figure 3d presents significant correlations between our predicted and experimental data, which are expressed here on a relative scale of differences between ancestral and minor alleles of a given SNP.

Considering all these robust correlations together, we can say that in this work, this application of our Web service SNP_TATA_Comparator [45] to study atherosclerosis in humans is valid and useful.

### 2.9. In Silico Validation of Our Predictions Based on Clinical SNP Markers as a Whole

After experimental verification of our predictions, next we compared them (using current dbSNP build No. 151 dated 2017 [12]) with those obtained with the previous build No. 147 of this database dated 2016 (i.e., almost four times fewer SNPs as compared to the current one) [12] to estimate the robustness of our method with respect to the annual growth of the SNP count.

In row 1 of Table 1, readers can see that the genome-wide norm is a greater number SNPs damaging the TBP-sites within human gene promoters (*n* ≤ 800) than those improving these sites (*n* ≥ 200). This genome-wide pattern was first noted empirically [62] and then confirmed significantly (*p* > 0.99, binomial distribution) using ChIP-seq data within the “1000 Genomes” project [63]. This observation allows us to estimate the cumulative effect of all the newly predicted candidate SNP markers at the genome-wide level in comparison with the clinical SNP markers used to predict them, as we have done in many studies [49,50,51,54] to verify in silico the reliability of our predictions.

Row 2 of this table shows the results of biomedical verification of our Web service SNP_TATA_Comparator using the clinically known SNP markers of diseases in TBP-sites that was conducted when the software was just created [45]. As one can see, its predictions fit the genome-wide norm shown by row 1 of Table 1, which means neutral drift of the majority of SNPs causing human diseases, in agreement with both Haldane’s dilemma [25] and neutral evolution theory [24].

Row 3 here presents the results of our previous article on the atherosclerosis-related candidate SNP markers identified using one of the previous builds (No. 147) of dbSNP [12]. These data seem to follow the same pattern, indeed. Finally, as presented in row 4 of Table 1, our current predictions (made here by means of current build No. 151 of dbSNP [12]) reproduce the same pattern. This observation proves the robustness of our approach with respect to the annual growth of the SNP count.

As readers can see, the three rightmost columns of rows 1–4 of Table 1 present the numbers of candidate SNP markers accelerating (*n*↑ = 91) and slowing (*n*↓ = 54) atherogenesis as well as an estimate of the statistical significance of their difference from one another according to binomial distribution (*p* < 0.01). Consequently, readers can see reliable predominance of candidate SNP markers of atherogenesis acceleration over those of its deceleration, which means that the above-mentioned neutral drift of this kind of human SNPs supports atherogenesis acceleration. This finding seems to be in line with one of the well-known gerontological health care strategies when in utero, in childhood, adolescence, and reproductive adulthood, the human body can successfully respond to deadly stressors (e.g., acute infection) by subthreshold slowly progressing pathologies (e.g., atherogenesis), with clinically over-threshold manifestations (e.g., stroke) seen only in the very old [157]. It is important that this observation is statistically significant here, whereas in our previous article [55], there was only a tendency for this pattern (i.e., *n*↑ =19, *n*↓ =16, *p* > 0.33). Therefore, the fourfold increase in the SNP count in the current build No. 151 of dbSNP [12] versus its previous build No. 147 allows us to see a new genome-wide pattern.

### 2.10. Human Genes Related to Mitochondrial Genome Integrity and Instability of the Atherosclerotic Plaque

Following both in vitro and in silico verification of our predictions, we finally applied our newly validated Web service to atherosclerosis-related research to investigate several human genes associated with the maintenance of mitochondrial genome integrity [56], which are currently researched widely to slow atherogenesis.

Human gene *POLG* encodes a catalytic subunit of DNA polymerase γ (hereinafter: without any clinically proven SNP markers of human diseases within TBP-sites of its promoters) and carries six and 23 unannotated SNPs corresponding to under- and overexpression of this gene (Table 8). Using the PubMed keyword search utility [38], we found an original research article [158] on POLG deficiency as a physiological marker of an elevated level of mitochondrial DNA damage, which accelerates atherogenesis and vice versa. That is why we proposed six and 23 candidate SNP markers of speeding up atherogenesis (e.g., rs1266453407:g) and an atherogenesis slowdown (e.g., rs776506626, see Figure 2e), respectively, as one can see in Table 8. Finally, in this case, due to a PubMed search [38], we learned that this sort of atherogenesis acceleration could be prevented using antioxidant MitoQ [159], whereas the aforementioned type of atherogenesis retardation can be attenuated by both a fructose-rich diet [160] and alcohol without green tea [161].

Human gene *PGC1A* (peroxisome proliferator-activated receptor γ coactivator 1α, synonym: PPARGC1A) contains two unannotated SNPs—rs1254748756 and rs1206245736—causing PGC1A downregulation as an atheroprotector [162]. Thus, they can be candidate SNP markers of retarded atherogenesis (Table 8). Finally, this gene has another pair of unannotated SNPs, rs772816414 and rs1334636034, increasing PGC1A expression, which seems atheroprotective too [163]. Accordingly, they can also be candidate SNP markers of an atherogenesis slowdown (Table 8).

Human gene *TFAM* (mitochondrial transcription factor A) carries seven unannotated SNPs reducing concentrations of this regulatory protein and as many unannotated SNPs upregulating it (Table 8). According to PubMed [38], in a murine model of a human disease, an adipose-tissue-specific deletion of TFAM is atheroprotective [164], whereas a TFAM excess is proatherogenic in a rat model of human atherosclerosis [165]. In addition, nutritionists have reported that chronic alcohol consumption upregulates TFAM as a proatherogenic risk factor [166]. All these independent observations allow us to suggest 14 candidate SNP markers of retarded (e.g., rs1349790536) and sped up (e.g., rs943871999) atherogenesis, which are listed in Table 8.

Human gene *ATM* has 14 and 16 unannotated SNPs, which can respectively decrease and increase the amount of the ATM serine/threonine kinase encoded by this gene (Table 8). Our keyword search through PubMed [38] yielded biomedical data on this enzyme’s excess as an atheroprotector [167], and there is an ATM-deficient murine model of human atherosclerosis [168,169,170,171,172]. With this in mind, we propose 14 and 16 candidate SNP markers enhancing (e.g., rs773550815:g) and weakening (e.g., rs773550815:t) atherogenesis (Table 8). In particular, this time using PubMed [38], we found nutritionists’ report that postprandial tea is an atheroprotector in contrast to proatherogenic effects of postprandial coffee [169] (Table 8).

Looking through Table 8, we did not find any neutral drift (row 5 of Table 1: *n*_<_ = 29 and *n*_>_ = 48 at *p* < 0.02) or predominance of candidate SNP markers accelerating atherogenesis (row 5 of Table 1: *n*_↑_ = 27 and *n*_↓_ = 50 at *p* < 0.01). By contrast, these patterns were significant in the case of local surroundings of clinical SNP markers of human diseases (Table 1, Table 2, Table 3, Table 4, Table 5, Table 6 and Table 7).

As an independent test of this discrepancy, we next similarly analyzed the same number of miRNA genes involved in another gene network of instability of the atherosclerotic plaque [57]. Let us consider what this test yielded (see Table 9).

Human gene *MIR10B* contains one unannotated SNP, rs1388274194, able to elevate the miR-10b level (Figure 2f), and another one, rs564940769, which can reduce it (Table 9). Next, a keyword search of PubMed [38] pointed to a biochemical research article [173] about an miR-10b deficit as an atheroprotector. That is why we recommend two candidate SNP markers (rs1388274194 and rs564940769) of speeding up and slowing down atherogenesis, respectively (Table 9).

Finally, in this case, in the PubMed database, we also learned that this sort of atherogenesis acceleration could be prevented using dietary supplements of berberine [174].

Human gene *MIR21* carries four unannotated SNPs of miR-21 downregulation, which is atheroprotective [175] according to PubMed [38]. This result allows us to suggest all of them as candidate SNP markers of retarded atherogenesis (e.g., rs752908264), as shown in Table 9.

Human gene *MIR143* carries four unannotated SNPs, which can cause its overexpression, whereas miR-143 upregulation in cells overloaded with cholesterol prevents their transformation into foam cells thereby slowing down atherogenesis [176,177,178], in line with our outcome of a keyword search in PubMed [38], as summed up in Table 9. On this basis, we predict four candidate SNP markers of an atherogenesis delay (e.g., rs369969688, Table 9). In addition, within the promoter of this gene, we recognized three unannotated SNPs (rs1369382070, rs568314295, and rs1033081876) causing miR-143 downregulation, which is a physiological marker of atherogenesis progression [179]. Thus, we classified them as candidate SNP markers of atherogenesis acceleration (Table 9).

Human gene *MIR145* contains one unannotated SNP, rs909856793, decreasing the miR-145 amount, and two unannotated SNPs (rs746241408 and rs778670319), which can elevate the miR-145 level, as readers can see in Table 9. Because of our keyword search in PubMed [38] resulted in a clinical cohort-based report [180] on an miR-145 excess as an atheroprotector and vice versa, we predicted rs909856793 to be a candidate SNP marker of speeding up atherogenesis and two candidate SNP markers (rs746241408 and rs778670319) of its slowdown (Table 9).

Row 6 of Table 1 summarizes our predictions here for the core promoters of the miRNA genes involved in the gene network of instability of the atherosclerotic plaque [57] (Table 9) in comparison with the protein-coding genes from the same gene network (Table 8). As readers can see, there is no neutral drift (row 6 of Table 1: *n*_<_ = 5 and *n*_>_ = 11 at *p* < 0.05) and no predominance of candidate SNP markers accelerating atherogenesis (row 6 of Table 1: *n*↑ = 2 and *n*↓ = 14 at *p* < 0.01). This means that the two independent predictions are in agreement that natural selection against underexpression of the human genes dealing with mitochondrial genome integrity [56] and atherosclerotic plaque instability [57] supports an atherogenesis slowdown.

## 3. Materials and Methods

### 3.1. DNA Sequences

We retrieved SNPs from the dbSNP database [12] and DNA sequences from the Ensembl database [11] in reference human genome assembly GRCh38/hg38 via the UCSC Genome Browser [13].

### 3.2. Analysis of DNA Sequences

We analyzed SNPs within DNA sequences using our previously created public Web service SNP_TATA_Comparator [45], which implements our model of three-step TBP-promoter binding, as described in depth within Supplementary Materials [181,182,183] (i.e., Section S1 “Supplementary DNA sequence analysis”).

### 3.3. Keyword Searches in the PubMed Database

**Figure 4:**
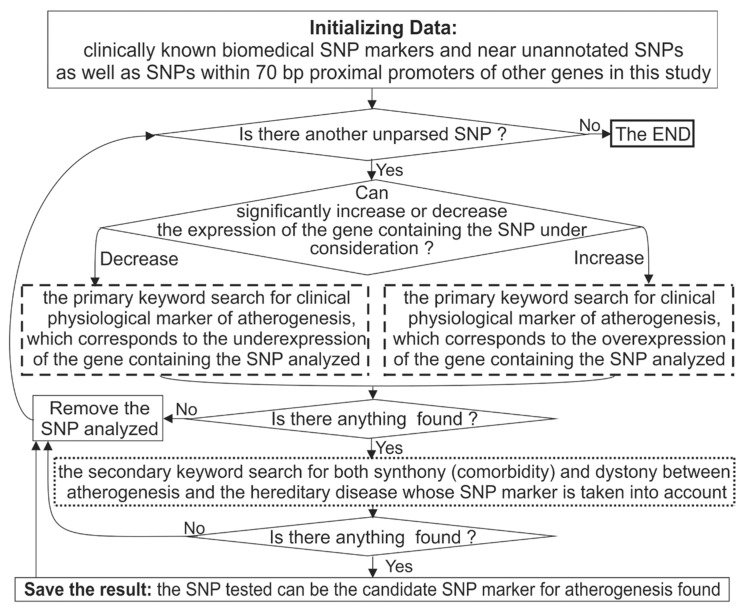
A flow chart of the keyword search for atherosclerosis as a comorbidity of hereditary diseases whose candidate SNP markers can alter TBP-sites in the human gene promoters.

For each candidate SNP marker predicted in this work, we performed a standard keyword search in the PubMed database [38] as illustrated in Figure 4.

### 3.4. In Vitro Measurements

For each of the five chosen candidate SNP markers of atherogenesis predicted here—*GCG*: rs183433761, *LEP*: rs201381696, *HBB*: rs34500389, *HBD*: rs35518301, and *F3*: rs563763767—using an electrophoretic mobility shift assay (EMSA), we experimentally measured absolute values of equilibrium dissociation constant K_D_ of TBP-promoter complexes, as described in detail within Supplementary Materials [184] (i.e., Section S2 “Supplementary in vitro measurement”).

### 3.5. Statistical Analysis

A comparison of our predictions with the experimental values of equilibrium dissociation constant K_D_ of TBP-promoter complexes was conducted using two options, “Multiple Regression” and “Nonparametrics,” in a standard toolbox, STATISTICA (Statsoft^TM^, Tulsa, USA).

## 4. Conclusions

Because TBP-binding regions are the best-studied regulatory sequences within the human genome [21], here, using SNP_TATA_Comparator [45], we analyzed 1189 SNPs within these regions and as a result predicted 237 candidate SNP markers of atherosclerosis in addition to the only clinically proven SNP marker of this disorder (Table 2, Table 3, Table 4, Table 5, Table 6, Table 7, Table 8 and Table 9) as shown in row 7 of Table 1.

This result increases targetability fivefold, and thus reduces the costs as compared to the traditional random heuristic selection of candidate SNP markers for clinical testing in patients and conventionally healthy volunteers (e.g., see [22]). Given that the regulatory and protein-coding regions of different transcripts can overlap with each other, any given SNP can simultaneously be a missense mutation, an intronic or 5′- or 3′-untranslated variant, or some other variant (e.g., rs370819229 and rs777650793). Thus, further stratification of the list of 238 candidate SNP markers predicted here is possible thanks to a number of public Web services (for review, see [18]).

Those atherosclerosis-related candidate SNP markers that can survive clinical examination—on cohorts of patients and healthy volunteers as whole-genome landmarks of this disorder—may become useful for physicians (may help to optimize treatment of a patient according to his/her individual sequenced genome) as well as for the general population (may help to choose a lifestyle slowing the inevitable atherogenesis).

Additionally, when the dbSNP database [12] includes the occurrence rates of minor alleles of SNPs in various human races and large ethnic groups (e.g., Slavs), in addition to such data already present in dbSNP for narrow aboriginal ethnic groups (e.g., Scotland scotlades) as migration-related SNP markers, the list of candidate SNP markers predicted by SNP_TATA_Comparator [45] can be stratified in this regard.

Unexpectedly, summing up all the above results, here we for the first time detected two statistically significant patterns in the genome: (1) neutral drift that leads to accelerated atherogenesis and (2) the direction of natural selection that impedes atherogenesis. Therefore, their superposition on each other stabilizes the total effect and thus establishes the norm of the trait of atherogenesis development.

Finally, it should be noted that the atherogenesis rate is a trait that can be controlled via adjustment of the lifestyle (e.g., diet), as unambiguously demonstrated in Table 2, Table 3, Table 4, Table 5, Table 6, Table 7, Table 8 and Table 9.

## Figures and Tables

**Table 5:** Atherogenesis-related candidate SNP markers within the gene promoters where SNPs of TBP-sites are clinically linked with obesity.

*Gene*	dbSNP ID [12] or [Ref]	DNA, Genome Sequence				K_D_, nM						Clinical Data or *Candidate SNP Markers*	AS	Ref.
		5′ flank	WT	min	3′ flank	WT	min	Δ	Z	α	ρ			
*GCG*	*rs183433761*	*gctggagagt*	*a*	*g*	*tataaaagca*	*1*	*2*	*< *	*8*	*10^−6^*	*A*	*hypogluca-gonemia and postprandial increased ratio [INS]:[GCG] accelerates atherogenesis*	↑	[115,116,117,118]
	*rs1342326265*	*tataaaagca*	*g*	*a*	*tgcgccttgg*	*0.9*	*1.2*	*< *	*5*	*10^−6^*	*A*		↑	
	*rs757035851*	*tatataaaag*	*cag*	*-*	*tgcgccttgg*	*0.9*	*1.1*	*< *	*3*	*10^−3^*	*B*		↑	
	*rs773542506*	*tggagagtat*	*a*	*g*	*taaaagcagt*	*1*	*2*	*< *	*14*	*10^−6^*	*A*		↑	
	*rs984859897*	*agctggagag*	*t*	*c*	*atataaaagc*	*1*	*2*	*< *	*11*	*10^−6^*	*A*		↑	
*LEP*	*rs201381696*	*tcgggccgct*	*a*	*g*	*taagaggggc*	*4*	*12*	*< *	*18*	*10^−6^*	*A*	*hypoleptine-mia, obesity*	↑	[119]
	*rs1458202641*	*ggggcgggca*	*a*	*a*	*gaggggcggg*	*4*	*6*	*< *	*8*	*10^−6^*	*A*	*accelerate atherogenesis*	↑	[120]
	*rs34104384*	*ccgctataag*	*a*	*t*	*ggggcgggca*	*4*	*3*	*> *	*4*	*10^−3^*	*B*	*slowed down atherogenesis (athero-protector)*	↓	[121]
	*rs200487063*	*tgatcgggcc*	*g*	*a*	*ctataagagg*	*4*	*2*	*> *	*6*	*10^−6^*	*A*		↓	
	*rs1249322424*	*gtgatcgggc*	*c*	*a*	*gctataagag*	*4*	*3*	*> *	*3*	*10^−2^*	*C*		↓	
*APOA1*	[122]	tgcagacata	a	c	ataggccctg	3	4	<	5	10^−6^	A	hematuria, fatty liver, obesity	↑	[122]
	*rs1428975217*	*acataaatag*	*g*	*t*	*ccctgcaaga*	*2.6*	*3.0*	*< *	*2*	*0.05*	*D*	*accelerated atherogenesis (exogenous APOA1 is atheropro-tector)*	↑	[123,124]
	*rs1017922094*	*cagacataaa*	*t*	*c*	*aggccctgca*	*3*	*6*	*< *	*9*	*10^−6^*	*A*		↑	
	*rs1297144980*	*cctggctgca*	*6bp*	*-*	*aataggccct*	*3*	*21*	*< *	*30*	*10^−6^*	*A*		↑	
*HTR2C*	rs3813929	cccctcatcc	c	t	gcttttggcc	73	56	>	6	10^−6^	A	obesity com-plication of olanzapine antipsycho-tic therapy	↑	[15]
	*rs1348095721*	*agagcgtggt*	*g*	*a*	*cagattcacc*	*73*	*56*	*> *	*5*	*10^−3^*	*B*	*hypercor-tisolemia accelerates atherogenesis that can be slowed down by lorcaserin*	↑	[125,126]
	*rs1444133212*	*caagagcgtg*	*c*	*t*	*caagagcgtg*	*73*	*39*	*> *	*13*	*10^−6^*	*A*		↑	
	*rs886838672*	*ccgcttttgg*	*c*	*t*	*ccaagagcgt*	*73*	*34*	*> *	*15*	*10^−6^*	*A*		↑	
	*rs1376972872*	*tcccgctttt*	*g*	*t*	*gcccaagagc*	*73*	*43*	*> *	*11*	*10^−6^*	*A*		↑	
	*rs1222709869*	*tggctcctcc*	*c*	*t*	*ctcatcccgc*	*73*	*50*	*> *	*8*	*10^−6^*	*A*		↑	
*IL1B*	rs1143627	ttttgaaagc	c	t	ataaaaacag	5	2	>	15	10^−6^	A	obesity, Gra-ves’ disease, depression; lung, liver, breast, and gastric cancer	↑	[127,128,129,130,131,132,133]
												*accelerated atherogenesis, *		[134]
	*rs549858786*	*tgaaagccat*	*a*	*t*	*aaaacagcga*	*5*	*7*	*<*	*8*	*10^−6^*	*A*	slowed down atherogenesis	↓	[135,136]

Genes: *GCG*, glucagon; *LEP*, leptin; *APOA1*, apolipoprotein A1; *HTR2C*, 5-hydroxytryptamine receptor 2C (synonym: serotonin receptor 2C); *IL1B*, interleukin 1β. Deletions, *APOA1*: 6bp = gacata.

**Table 6:** Atherogenesis-related candidate SNP markers within the gene promoters where SNPs of TBP-sites are clinically linked with cancers.

Gene	dbSNP ID [12] or [Ref]	DNA, Genome Sequence				K_D_, nM						Clinical Data or Candidate SNP Markers	AS	[Ref]
		5′ flank	WT	min	3′ flank	WT	min	Δ	Z	α	ρ			
*ADH7*	rs17537595	gctgctgtta	t	c	atacaacaga	1	3	<	13	10^−6^	A	esophageal cancer	↑	[137]
	*rs372329931*	*agctgctgtt*	*a*	*g*	*tatacaacag*	*1*	*3*	*<*	*13*	*10^−6^*	*A*	*post-esopha-gectomy necrosis comorbid with atherogenesis*	↑	[138]
	*rs755152695*	*caagctgctg*	*t*	*c*	*tatatacaac*	*1.0*	*1.4*	*<*	*4*	*10^−3^*	*B*		↑	
	*rs1238877951*	*gcacaagctg*	*c*	*a*	*tgttatatac*	*1.0*	*1.2*	*<*	*3*	*10^−2^*	*C*		↑	
*HSD17B1*	rs201739205	aggtgatatc	a	c	agcccagagc	13	18	<	5	10^−3^	B	breast cancer	↓	[139]
	*rs748743528*	*gcaggtgata*	*t*	*c*	*caagcccaga*	*13*	*28*	*<*	*13*	*10^−6^*	*A*	*biomarker of the atherogenesis slowed down by drug IMM-H007*	↓	[140]
	*rs779674159*	*agcaggtgat*	*a*	*t*	*tcaagcccag*	*13*	*35*	*<*	*18*	*10^−6^*	*A*		↓	
	*rs1282820277*	*cgaagcaggt*	*g*	*a*	*atatcaagcc*	*13*	*7*	*>*	*9*	*10^−6^*	*A*	*accelerated atherogenesis*	↑	[141]
	*rs755636251*	*ggcgaagcag*	*g*	*t*	*tgatatcaag*	*13*	*11*	*>*	*2*	*0.05*	*D*		↑	
	*rs1332869256*	*gggcgaagca*	*g*	*c*	*gtgatatcaa*	*13*	*11*	*>*	*4*	*10^−3^*	*B*		↑	
*MLH1*	rs63750527	tacaacaaag	g	c	ggacttcaga	11	9	>	4	10^−3^	B	nonpolyposis colon cancer	↓	[15]
	rs756099600	atacaacaaa	g	a, c	gggacttcag	11	10	>	3	0.05	D		↓	
	*rs753671152*	*caaaggggac*	*t*	*a*	*tcagaaatgt*	*11*	*9*	*>*	*4*	*10^−3^*	*B*	*improved DNA mismatch repair can slow down atherogenesis*	↓	[142,143,144]
	*rs1424963586*	*gatacaacaa*	*a*	*c*	*ggggacttca*	*11*	*9*	*>*	*3*	*10^−2^*	*C*		↓	
	*rs752622244*	*cttggagggg*	*g*	*t*	*atacaacaaa*	*11*	*3*	*>*	*17*	*10^−6^*	*A*		↓	
	*rs587778905*	*gcttggaggg*	*21bp*	*taaa*	*agaaatgtca*	*11*	*19*	*<*	*9*	*10^−6^*	*A*	*accelerated atherogenesis*, *which can be slowed down by restricting alcohol and red meat intake*	↑	
	*rs34285587*	*gggggataca*	*a*	g^*^	*caaaggggac*	*11*	*20*	*<*	*9*	*10^−6^*	*A*		↑	
	*rs864622145*	*ggagggggat*	*a*	*g*	*caacaaaggg*	*11*	*27*	*<*	*17*	*10^−6^*	*A*		↑	
*RET*	rs10900296	gccggcgctt	a	g, c	cctcgcttca	30	90	<	20	10^−6^	A	pheochro-mocytoma	↑	[15]
	*rs551321384*	*agccggcgct*	*t*	*c*	*acctcgcttc*	*30*	*75*	*<*	*17*	*10^−6^*	*A*	*dysregulated atheroprotector pentraxin-3*	↑	[145]
	*rs1191017949*	*cagccggcgc*	*t*	*c*	*tacctcgctt*	*30*	*48*	*<*	*7*	*10^−6^*	*A*		↑	
	*rs1237152255*	*tacctcgctt*	*c*	*t*	*ttacctcgct*	*30*	*20*	*>*	*6*	*10^−6^*	*A*	*slowed down atherogenesis*	↓	
	*rs1372293149*	*cgcagccggc*	*g*	*a*	*cttacctcgc*	*30*	*16*	*>*	*10*	*10^−6^*	*A*		↓	
	rs10900297	gcgcttacct	c	a	gcttcagtcc	30	9	>	17	10^−6^	A	pheochro-mocytoma	↓	[15]
*ESR2*	rs35036378	cctctcggtc	t	g	ttaaaaggaa	6	8	<	5	10^−3^	B	ESR2-deficient pT1 tumor	↑	[146]
	*rs766797386*	*ttaaaaggaa*	*g*	*t*	*aaggggctta*	*6*	*7*	*<*	*3*	*10^−2^*	*C*	*accelerated calcification*	↑	[147,148]
*DHFR*	rs10168	ctgcacaaat	g	a	gggacgaggg	15	9	>	9	10^−6^	A	resistance to methotrexate	↓	[149]
	*rs750793297*	*tgcacaaatg*	*g*	*t*	*ggacgagggg*	*15*	*13*	*>*	*3*	*10^−2^*	*C*	*DHFR deficit is a biomarker of accelerated atherogenesis*, *which can be slowed down by smoking restriction*	↓	[150,151]
	*rs1464445339*	*ggggcggggc*	*35bp*	*-*	*ggccacaatt*	*15*	*170*	*<*	*44*	*10^−6^*	*A*		↑	
	*rs766799008*	*gcctgcacaa*	*a*	*g*	*tggggacgag*	*15*	*19*	*<*	*3*	*10^−3^*	*B*		↑	
	*rs764508464*	*gcctgcacaa*	*a*	*-*	*tggggacgag*	*15*	*37*	*<*	*17*	*10^−6^*	*A*		↑	
	*rs754122321*	*ctcgcctgca*	*c*	*g*	*aaatggggac*	*15*	*25*	*<*	*9*	*10^−6^*	*A*		↑	
	*rs1328822484*	*cctcgcctgc*	*a*	*g*	*caaatgggga*	*15*	*58*	*<*	*25*	*10^−6^*	*A*		↑	

Genes: *ADH7*, alcohol dehydrogenase 7; *HSD17B1*, hydroxysteroid (17-β) dehydrogenase 1; *MLH1*, DNA mismatch repair protein Mlh1; *RET*, Ret proto-oncogene; *ESR2*, estrogen receptor 2 (β); *DHFR*, dihydrofolate reductase. Deletions, *MLH1*: 21bp = ggatacaacaaaggggacttc; *DHFR*: 35bp = ctcgcctgcacaaatggggacgaggggggcggggc.

**Table 7:** Atherogenesis-related candidate SNP markers within the gene promoters in which SNPs of TBP-sites are clinically linked with developmental disorders.

Gene	dbSNP ID [12] or [Ref]	DNA, Genome Sequence				K_D_, nM						Clinical Data or Candidate SNP Markers	AS	Ref.
		5′ flank	WT	min	3′ flank	WT	min	Δ	Z	α	ρ			
COMT	rs370819229	ccgcccgcca	c	a*	ggcctgcgtc	187	211	<	2	0.05	D	dilated car-diomyopathy	↓	[15]
	rs901020754	acggcctgcg	t	c	ccgccaccgg	187	243	<	5	10^−3^	B	slowed atherogenesis due to both estradiol (substrate) and 2-metho-xy estradiol (metabolite), which are athero-protective	↓	[152]
	rs779542396	cgccacggcc	t	c	gcgtccgcca	187	243	<	5	10^−3^	B		↓	
	rs45593642	ccaccggaag	c	a	gccctcctaa	187	115	>	9	10^−6^	A		↓	
	rs45581136	gccaccggaa	g	a	cgccctccta	187	160	>	3	10^−2^	C		↓	
	rs868447575	cgtccgccac	c	a, t	ggaagcgccc	187	74	>	17	10^−6^	A		↓	
	rs1369731401	gcgtccgcca	c	a	cggaagcgcc	187	118	>	8	10^−6^	A		↓	
	rs928358205	ctgcgtccgc	c	t	accggaagcg	187	82	>	13	10^−6^	A		↓	
	rs1060501404	gcctgcgtcc	g	a	ccaccggaag	187	137	>	5	10^−6^	A		↓	
	rs1296549321	ggcctgcgtc	c	t	gccaccggaa	187	152	>	4	10^−3^	B		↓	
	rs1333679310	cacggcctgc	g	a	tccgccaccg	187	110	>	10	10^−6^	A		↓	
	rs981175339	ccacggcctg	c	a, t	cgtccgccac	187	113	>	9	10^−6^	A		↓	
	rs748298389	gccacggcct	g	t	cgtccgccac	187	70	>	18	10^−6^	A		↓	
	rs1428300695	ccgccacggc	c	t*	tgcgtccgcc	187	117	>	9	10^−6^	A		↓	
	rs562298402	cgcccgccac	g	a	gcctgcgtcc	187	150	>	4	10^−3^	B		↓	
	rs1249101844	cgcaccccgc	15bp	-	ccgccaccgg	187	83	>	15	10^−6^	A		↓	
	rs777650793	gtccgccacc	g	a	gaagcgccct	187	82	>	15	10^−6^	A	cardiovascu-lar disease	↓	[15]
TGFBR2	rs138010137	cgcagcgctg	a	g	gttgaagttg	29	39	<	6	10^−6^	A	aortic thora-cic aneurysm & dissection	↑	[15]
	rs1300366819	cgctgagttg	a	g	agttgagtga	29	53	<	16	10^−6^	A	unchecked T-cell activation accelerates atherogenesis	↑	[153]
	rs1310294304	agcgctgagt	t	a	gaagttgagt	29	11	>	17	10^−6^	A	hypertension accelerates atherogenesis	↑	[154]
FGFR2	rs886046768	agagcgcggt	g	a	gagagccgag	115	31	>	22	10^−6^	A	cranio-synostosis	↑	[15]
	rs1212347974	tggaggagag	c	t	gcggtggaga	115	99	>	3	10^−2^	C	accelerated atherogenesis that can be slowed down by heparin-derived oligo-saccharide	↑	[155,156]
	rs1027484343	ctggaggaga	g	t*	cgcggtggag	115	101	>	3	10^−2^	C		↑	
	rs1226640384	ggctggagga	g	c	agcgcggtgg	115	99	>	3	10^−2^	C		↑	
	rs1189849606	gcggctggag	g	a	agagcgcggt	115	48	>	17	10^−6^	A		↑	
	rs971411400	atggtggtaa	c	t*	agtcatcctg	13	11	>	3	10^−2^	C		↑	
	rs778187292	cctgtatggt	g	a	gtaacagtca	13	7	>	9	10^−6^	A		↑	
	rs1377663539	gggcggcggc	t	c	ggaggagagc	115	139	<	3	10^−2^	C	slowed atherogenesis	↑	
	rs751951199	aatcgcctgt	a	g	tggtggtaac	13	30	<	13	10^−6^	A		↑	
	rs757648006	tcttaatcgc	c	g	tgtatggtgg	13	17	<	3	10^−2^	C		↑	
	rs387906677	atcgcctgta	t	g	ggtggtaaca	13	27	<	11	10^−6^	A	bent bone dysplasia	↑	[15]
													↑	

Genes: *COMT*, catechol-O-methyltransferase; *TGFBR2*, transforming growth factor beta receptor 2; *FGFR2*, fibroblast growth factor receptor 2 (synonyms: keratinocyte growth factor receptor, bacteria-expressed kinase). Deletions, *COMT*: 15bp = ccgccacggcctgcg.

**Table 8:** Atherogenesis-related candidate SNP markers within promoters of the human protein-coding genes related to the maintenance of mitochondrial genome integrity.

Gene	dbSNP ID [12] or [Ref]	DNA, Genome Sequence				K_D_, nM						Clinical Data or Candidate SNP Markers	AS	Ref.
		5′ flank	WT	min	3′ flank	WT	min	Δ	Z	α	ρ			
*POLG*	*rs776506626*	*gaagcgctgg*	*a*	*g*	*tatttaccag*	*5*	*8*	*<*	*6*	*10^−6^*	*A*	*accelerated atherogenesis*, *which can be slowed down by antioxidant MitoQ*	↑	[158,159,160,161]
	*rs142347031*	*gagcgcttac*	*t*	*g*	*aatgcagttt*	*6*	*13*	*<*	*13*	*10^−6^*	*A*		↑	
	*rs888362291*	*ggacaccatg*	*a*	*c*	*gcatgcacat*	*41*	*53*	*<*	*5*	*10^−3^*	*B*		↑	
	*rs1460331646*	*cctggacacc*	*a*	*g*	*tgagcatgca*	*41*	*53*	*<*	*5*	*10^−3^*	*B*		↑	
	*rs760935752*	*cgtttcctgg*	*a*	*g*	*caccatgagc*	*41*	*53*	*<*	*5*	*10^−3^*	*B*		↑	
	*rs1266453407:g*	*ttggcggccc*	*t*	*g*	*cctattggtc*	*24*	*30*	*<*	*3*	*10^−3^*	*B*		↑	
	*rs1266453407:a*	*ttggcggccc*	*t*	*a*	*cctattggtc*	*24*	*20*	*>*	*3*	*10^−2^*	*C*	*slowed atherogenesis*, *while fructose-rich diet can accelerate mitochondrial DNA damage as atherogenic nutrition*, *as can alcohol*, *the effect of which could be reversed by green tea*	↓	
	*rs1257127556*	*ccctcctatt*	*g*	*a*	*gtcgcgctgg*	*24*	*5*	*>*	*22*	*10^−6^*	*A*		↓	
	*rs1210506140*	*gccctcctat*	*t*	*a*	*ggtcgcgctg*	*24*	*8*	*>*	*16*	*10^−6^*	*A*		↓	
	*rs3176154*	*tggcggccct*	*c*	*t*	*ctattggtcg*	*24*	*14*	*>*	*9*	*10^−6^*	*A*		↓	
	*rs1024469208*	*cctcacagac*	*c*	*t*	*tcggcccctg*	*35*	*29*	*>*	*3*	*10^−2^*	*C*		↓	
	*rs747270023*	*cccccaccct*	*c*	t *	*acagacctcg*	*35*	*15*	*>*	*13*	*10^−6^*	*A*		↓	
	*rs2307434*	*cccccaccct*	*c*	*-*	*acagacctcg*	*35*	*21*	*>*	*8*	*10^−6^*	*A*		↓	
	*rs983016336*	*cttcccccac*	*c*	*a*	*ctcacagacc*	*35*	*25*	*>*	*6*	*10^−6^*	*A*		↓	
	*rs1318374817*	*tgattgtctt*	*c*	*a*	*ccccaccctc*	*35*	*22*	*>*	*9*	*10^−6^*	*A*		↓	
	*rs768707883*	*gctcctgatt*	*g*	*a*	*tcttccccca*	*35*	*20*	*>*	*9*	*10^−6^*	*A*		↓	
	*rs974500502*	*tctcctgctc*	*c*	*t*	*tgattgtctt*	*35*	*23*	*>*	*8*	*10^−6^*	*A*		↓	
	*rs142460560*	*tgaagcgctg*	*g*	*t*	*atatttacca*	*5*	*3*	*>*	*7*	*10^−6^*	*A*		↓	
	*rs769410130*	*cagggctaag*	*c*	t *	*agcttccagc*	*41*	*9*	*>*	*23*	*10^−6^*	*A*		↓	
	*rs749799663*	*ggccatctca*	*g*	*a*	*ggctaagcag*	*41*	*33*	*>*	*4*	*10^−3^*	*B*		↓	
	*rs769104909*	*acatggccat*	*c*	*a*	*tcagggctaa*	*41*	*16*	*>*	*16*	*10^−6^*	*A*		↓	
	*rs1309549222*	*gcatgcacat*	*g*	*a*	*gccatctcag*	*41*	*17*	*>*	*15*	*10^−6^*	*A*		↓	
	*rs767895645*	*ccatgagcat*	*g*	a *	*cacatggcca*	*41*	*10*	*>*	*20*	*10^−6^*	*A*		↓	
	*rs144068243*	*acaccatgag*	*c*	*t*	*atgcacatgg*	*41*	*17*	*>*	*18*	*10^−6^*	*A*		↓	
	*rs1206132846*	*gtttcctgga*	*c*	*t*	*accatgagca*	*41*	*28*	*>*	*7*	*10^−6^*	*A*		↓	
	*rs766414821*	*gcatgcgttt*	*c*	*t*	*ctggacacca*	*41*	*35*	*>*	*3*	*10^−2^*	*C*		↓	
	*rs1333954036*	*tggtgttcga*	*c*	*t*	*gtggaggtct*	*62*	*36*	*>*	*7*	*10^−6^*	*A*		↓	
	*rs953495957*	*gggggaggcc*	*g*	*a*, *t*	*tacccgtggc*	*62*	*43*	*>*	*5*	*10^−3^*	*B*		↓	
	*rs961105910*	*tccagccagt*	*a*	*t*	*aaagaagcca*	*16*	*7*	*>*	*11*	*10^−6^*	*A*		↓	
*PGC1A*	*rs1254748756*	*ggactgtagt*	*a*	*g*	*agacaggtgc*	*5*	*18*	*<*	*18*	*10^−6^*	*A*	*slowed atherogenesis*	↓	[162]
	*rs1206245736*	*tgtttggatg*	*t*	*c*	*gtaaatgcag*	*5*	*10*	*<*	*10*	*10^−6^*	*A*		↓	
	*rs772816414*	*gtttggatgt*	*g*	*a*	*taaatgcagg*	*5*	*2*	*>*	*17*	*10^−6^*	*A*		↓	[163]
	*rs1334636034*	*gtgtttggat*	*g*	*a*	*tgtaaatgca*	*5*	*4*	*>*	*3*	*10^−2^*	*B*		↓	
*TFAM*	rs1349790536	gcccccatct	a	t	ccgaccggat	*18*	*25*	*<*	*5*	*10^−6^*	*A*	*slowed atherogenesis*, *while chronic alcohol intake upregulates TFAM as pro-atherogenic risk factor*	*↓*	[164,166]
	rs1442727766	cccgccccca	t	g	ctaccgaccg	*18*	*25*	*<*	*5*	*10^−6^*	*A*		*↓*	
	rs756889032	catgtggggc	g	t *	tgctgagtgc	*39*	*44*	*<*	*2*	*0.05*	*D*		*↓*	
	rs200473819	ctccgaagca	t	c	gtggggcgtg	*39*	*50*	*<*	*5*	*10^−3^*	*B*		*↓*	
	rs1247582808	tctccgaagc	a	g	tgtggggcgt	*39*	*50*	*<*	*5*	*10^−3^*	*B*		*↓*	
	rs1468597986	tcccgttact	a	g	tttctgaact	*5*	*11*	*<*	*9*	*10^−6^*	*A*		*↓*	
	rs1036018716	cctctcccgt	t	c	actatttctg	*5*	*11*	*<*	*10*	*10^−6^*	*A*		*↓*	
	rs943871999	cccatctacc	g	a	accggatgtt	*18*	*14*	*>*	*4*	*10^−3^*	*B*	*propensity to intimal thickening of an artery or vein related to accelerated atherogenesis*	*↑*	[165]
	rs571704530	ccccatctac	c	t	gaccggatgt	*18*	*13*	*>*	*5*	*10^−3^*	*B*		*↑*	
	rs911015016	cccccatcta	c	t	cgaccggatg	*18*	*15*	*>*	*3*	*10^−2^*	*C*		*↑*	
	rs1355238441	tccgaagcat	g	a	tggggcgtgc	*39*	*13*	*>*	*18*	*10^−6^*	*A*		*↑*	
	rs1350851808	tttctccgaa	g	c	catgtggggc	*39*	*34*	*>*	*3*	*0.05*	*D*		*↑*	
	rs1448492458	gatggcgttt	c	g	tccgaagcat	*39*	*30*	*>*	*4*	*10^−3^*	*B*		*↑*	
	rs1337389336	gagcgatggc	g	a *	tttctccgaa	*39*	*32*	*>*	*4*	*10^−3^*	*B*		*↑*	
*ATM*	rs540204119	cgggagtagg	t	c	agctgcgtgg	*12*	*15*	*<*	*3*	*10^−2^*	*C*	*accelerated atherogenesis*, *which can be slowed down by anti-malarial drug chloroquine when p53 expression is normal*, *as can mitochondria-targeted antioxidant MitoQ regardless of p53 level*	*↑*	[167,168,169,170,171,172]
	rs960185644	aagcgggagt	a	c	ggtagctgcg	*12*	*15*	*<*	*3*	*10^−2^*	*C*		*↑*	
	rs758371056	gtatttagta	c	t	ttttagtcag	*3*	*5*	*<*	*7*	*10^−6^*	*A*		*↑*	
	rs1424898053	tctctcgtat	t	c	tagtactttt	*3*	*5*	*<*	*8*	*10^−6^*	*A*		*↑*	
	rs1418169685	ctctctcgta	t	c	ttagtacttt	*3*	*4*	*<*	*4*	*10^−3^*	*B*		*↑*	
	rs1439694580	tgatctctct	c	g	tcgtatttag	*3*	*5*	*<*	*4*	*10^−3^*	*B*		*↑*	
	rs1461551448	cgggtccaat	a	c	accctccatc	*23*	*45*	*<*	*12*	*10^−6^*	*A*		*↑*	
	rs1479937619	agccgggtcc	a	g	ataaccctcc	*23*	*29*	*<*	*4*	*10^−3^*	*B*		*↑*	
	rs1179356361	ccagcatagc	c	t	gggtccaata	*23*	*26*	*<*	*2*	*0.05*	*D*		*↑*	
	rs1290688759	ttcacagata	t	c	aaaatattaa	*3*	*5*	*<*	*12*	*10^−6^*	*A*		*↑*	
	rs778072373	gttcacagat	a	g	taaaatatta	*3*	*5*	*<*	*10*	*10^−6^*	*A*		*↑*	
	rs1162474448	aacggaagtt	a	g	atatgatcat	*6*	*14*	*<*	*12*	*10^−6^*	*A*		*↑*	
	rs951064054	gccgcggttg	a	c	tactactttg	*6*	*17*	*<*	*13*	*10^−6^*	*A*		*↑*	
	rs773550815:g	gccgggtcca	a	g	taaccctcca	*23*	*33*	*<*	*7*	*10^−6^*	*A*		*↑*	
	rs773550815:t	gccgggtcca	a	t	taaccctcca	*23*	*21*	*>*	*2*	*0.05*	*D*	*slowed atherogenesis*, *while caffeine (ATM inhibi-tor) can post-prandially in-crease arterial stiffness for up to 3 h per cup of coffee as pro-atherogenic factor unlike mixtures of caffeine & catechins in tea as athero-protectors*	*↓*	
	rs976385260	acgacgaggg	c	a *	gaagagggtg	*69*	*50*	*>*	*6*	*10^−6^*	*A*		*↓*	
	rs1440912817	gacgacgagg	g	t	cgaagagggt	*69*	*51*	*>*	*6*	*10^−6^*	*A*		*↓*	
	rs1382260245	ggaggacgac	g	a	agggcgaaga	*69*	*36*	*>*	*12*	*10^−6^*	*A*		*↓*	
	rs942361620	gcggggagga	c	t	gacgagggcg	*69*	*49*	*>*	*6*	*10^−6^*	*A*		*↓*	
	rs1457610144	gggcggggag	g	a	acgacgaggg	*69*	*61*	*>*	*2*	*0.05*	*D*		*↓*	
	rs973002497	agacttggag	15bp	-	gggcggggag	*69*	*56*	*>*	*4*	*10^−3^*	*B*		*↓*	
	rs1222755392	ggcggggatg	a	t	ggagggcggg	*69*	*53*	*>*	*5*	*10^−3^*	*B*		*↓*	
	rs1242858005	ggaggggcgg	g	a	gatgaggagg	*69*	*57*	*>*	*4*	*10^−3^*	*B*		*↓*	
	rs1235571571	gcgggagtag	g	a	tagctgcgtg	*12*	*7*	*>*	*7*	*10^−6^*	*A*		*↓*	
	rs1254449321	agcgggagta	g	a	gtagctgcgt	*12*	*8*	*>*	*6*	*10^−6^*	*A*		*↓*	
	rs763320013	ggtccaataa	c	t	cctccatccc	*23*	*19*	*>*	*4*	*10^−3^*	*B*		*↓*	
	rs373553992	tccttctgtc	c	t	agcatagccg	*23*	*16*	*>*	*4*	*10^−3^*	*B*		*↓*	
	rs1397272250	gacgttcaca	g	t	atataaaata	*3*	*1*	*>*	*10*	*10^−6^*	*A*		*↓*	
	rs1401830174	tcgaaacagt	c	t	ataacggaag	*6*	*4*	*>*	*3*	*10^−2^*	*C*		*↓*	
	rs1310604038	ccaccgccgc	g	c, t	gttgatacta	*6*	*5*	*>*	*2*	*0.05*	*D*		*↓*	

Genes: *POLG*, catalytic subunit of DNA polymerase γ; *PGC1A*, peroxisome proliferator-activated receptor γ coactivator 1α (synonym: PPARGC1A); *TFAM*, mitochondrial transcription factor A; *ATM*, ATM serine/threonine kinase. Deletions, *ATM*: 15bp = gggcggggatgagga.

**Table 9:** Atherogenesis-related candidate SNP markers within the promoters of miRNA genes associated with instability of the atherosclerotic plaque.

Gene	dbSNP ID [12] or [Ref]	DNA, Genome Sequence				K_D_, nM						Clinical Data or Candidate SNP Markers	AS	Ref.
		5′ flank	WT	min	3′ flank	WT	min	Δ	Z	α	ρ			
*MIR10B*	*rs1388274194*	*cagcgaccta*	*g*	*t*	*cagcgaccta*	*14*	*4*	*>*	*14*	*10^−6^*	*A*	*ABCA1 over-inhibition that accelerates atherogenesis*, *which can be slowed down by dietary supplements of berberine*	↑	[173,174]
	*rs564940769*	*cggcagcgac*	*c*	g *	*taggtacctc*	*14*	*25*	*<*	*8*	*10^−6^*	*A*	*slowed atherogenesis*	↓	
*MIR21*	*rs752908264*	*gtgacatctc*	*c*	*t*	*atggctgtac*	*33*	*17*	*>*	*11*	*10^−6^*	*A*	*reduced calcification of atherosclerotic plaque*	↓	[175]
	*rs781007656*	*atcgtgacat*	*c*	*a*	*tccatggctg*	*33*	*10*	*>*	*20*	*10^−6^*	*A*		↓	
	*rs368618785*	*ctaccatcgt*	*g*	*t*	*acatctccat*	*33*	*10*	*>*	*20*	*10^−6^*	*A*		↓	
	*rs772840282*	*actgtctgct*	*t*	*a*	*gttttgccta*	*33*	*22*	*>*	*7*	*10^−6^*	*A*		↓	
*MIR143*	*rs369969688*	*cctctaacac*	*c*	*t*	*ccttctcctg*	*17*	*13*	*>*	*5*	*10^−3^*	*B*	*prevented transformation of cells over-loaded with cholesterol in-to foam cells slows down atherogenesis*	↓	[176,177,178]
	*rs142037609*	*tcccctctaa*	*c*	*t*	*accccttctc*	*17*	*5*	*>*	*17*	*10^−6^*	*A*		↓	
	*rs1035277319*	*caggtcccct*	*c*	*a*	*taacacccct*	*17*	*5*	*>*	*16*	*10^−6^*	*A*		↓	
	*rs999653534*	*ccaggtcccc*	*t*	*a*	*ctaacacccc*	*17*	*14*	*>*	*3*	*10^−2^*	*C*		↓	
	*rs1369382070*	*caccccttct*	*-*	*c*	*cctggccagg*	*17*	*22*	*<*	*5*	*10^−3^*	*B*	*accelerated atherogenesis*	↑	[179]
	*rs568314295*	*ccctctaaca*	*c*	*g*, *t*	*cccttctcct*	*17*	*21*	*<*	*3*	*10^−3^*	*B*		↑	
	*rs1033081876*	*cccctctaac*	*a*	*c*	*ccccttctcc*	*17*	*44*	*<*	*16*	*10^−6^*	*A*		↑	
*MIR145*	*rs909856793*	*cagctggtcc*	*t*	*c*	*tagggacacg*	*45*	*56*	*<*	*3*	*10^−3^*	*B*	accelerated atherogenesis	↑	[177,178,180]
	*rs746241408*	*cttagggaca*	*c*	*a*, *t*	*ggcggccttg*	*45*	*38*	*>*	*3*	*0.05*	*D*	*prevented transformation of cells over-loaded with cholesterol in-to foam cells slows down atherogenesis*	↓	
	*rs778670319*	*tccttaggga*	*c*	*t*	*acggcggcct*	*45*	*32*	*>*	*5*	*10^−6^*	*D*		↓	

## Supplementary Materials

Supplementary Materials can be found at https://www.mdpi.com/1422-0067/21/3/1045/s1.

## Abbreviations

EMSA	electrophoretic mobility shift assay
TBP	TATA-binding protein
SNP	single-nucleotide polymorphism
WHO	World Health Organization

## References

[1] Barquera S., Pedroza-Tobias A., Medina C., Hernandez-Barrera L., Bibbins-Domingo K., Lozano R., Moran A.E. (2015). Global overview of the epidemiology of atherosclerotic cardiovascular disease. Arch. Med. Res..

[2] Libby P., Buring J., Badimon L., Hansson G., Deanfield J., Bittencourt M., Tokgozoglu L., Lewis E. (2019). Atherosclerosis. Nat. Rev. Dis. Primers.

[3] Li A.C., Glass C.K. (2002). The macrophage foam cell as a target for therapeutic intervention. Nat. Med..

[4] Lusis A.J. (2000). Atherosclerosis. Nature.

[5] Glass C.K., Witztum J.L. (2001). Atherosclerosis: The road ahead. Cell.

[6] Hirayama S., Soda S., Ito Y., Matsui H., Ueno T., Fukushima Y., Ohmura H., Hanyu O., Aizawa Y., Miida T. (2010). Circadian change of serum concentration of small dense LDL-cholesterol in type 2 diabetic patients. Clin. Chim. Acta.

[7] Lathe R., Sapronova A., Kotelevtsev Y. (2014). Atherosclerosis and Alzheimer--diseases with a common cause? Inflammation, oxysterols, vasculature. BMC Geriatr..

[8] Napoli C., D’Armiento F., Mancini F., Postiglione A., Witztum J., Palumbo G., Palinski W. (1997). Fatty streak formation occurs in human fetal aortas and is greatly enhanced by maternal hypercholesterolemia. Intimal accumulation of low density lipoprotein and its oxidation precede monocyte recruitment into early atherosclerotic lesions. J. Clin. Investig..

[9] Trovato G.M. (2014). Sustainable medical research by effective and comprehensive medical skills: Overcoming the frontiers by predictive, preventive and personalized medicine. EPMA J..

[10] Telenti A., Pierce L., Biggs W., di Iulio J., Wong E., Fabani M., Kirkness E., Moustafa A., Shah N., Xie C. (2016). Deep sequencing of 10,000 human genomes. Proc. Natl. Acad. Sci. USA.

[11] Zerbino D., Wilder S., Johnson N., Juettemann T., Flicek P. (2015). The Ensembl regulatory build. Genome Biol..

[12] Sherry S., Ward M., Kholodov M., Baker J., Phan L., Smigielski E., Sirotkin K. (2001). dbSNP: The NCBI database of genetic variation. Nucleic Acids Res..

[13] Haeussler M., Raney B., Hinrichs A., Clawson H., Zweig A., Karolchik D., Casper J., Speir M., Haussler D., Kent W. (2015). Navigating protected genomics data with UCSC Genome Browser in a box. Bioinformatics.

[14] Wu J., Wu M., Li L., Liu Z., Zeng W., Jiang R. (2016). dbWGFP: A database and web server of human whole-genome single nucleotide variants and their functional predictions. Database.

[15] Landrum M., Lee J., Riley G., Jang W., Rubinstein W., Church D., Maglott D. (2014). ClinVar: Public archive of relationships among sequence variation and human phenotype. Nucleic Acids Res..

[16] Amberger J., Bocchini C., Schiettecatte F., Scott A., Hamosh A. (2015). OMIM.org: Online Mendelian Inheritance in Man (OMIM^®^), an online catalog of human genes and genetic disorders. Nucleic Acids Res..

[17] Mitsuyasu H., Izuhara K., Mao X., Gao P., Arinobu Y., Enomoto T., Kawai M., Sasaki S., Dake Y., Hamasaki N. (1998). Ile50Val variant of IL4R alpha upregulates IgE synthesis and associates with atopic asthma. Nat. Genet..

[18] Deplancke B., Alpern D., Gardeux V. (2016). The genetics of transcription factor DNA binding variation. Cell.

[19] Savinkova L., Ponomarenko M., Ponomarenko P., Drachkova I., Lysova M., Arshinova T., Kolchanov N. (2009). TATA box polymorphisms in human gene promoters and associated hereditary pathologies. Biochemistry.

[20] Mogno I., Vallania F., Mitra R.D., Cohen B. (2010). TATA is a modular component of synthetic promoters. Genome Res..

[21] Ponomarenko M., Mironova V., Gunbin K., Savinkova L., Maloy S., Hughes K. (2013). Hogness Box. Brenner’s Encyclopedia of Genetics.

[22] Varzari A., Deyneko I., Tudor E., Turcan S. (2016). Polymorphisms of glutathione S-transferase and methylenetetrahydrofolate reductase genes in Moldavian patients with ulcerative colitis: Genotype-phenotype correlation. Meta Gene.

[23] Pocai B. (2019). The ICD-11 has been adopted by the World Health Assembly. World Psychiatry.

[24] Kimura M. (1968). Evolutionary rate at the molecular level. Nature.

[25] Haldane J.B.S. (1957). The cost of natural selection. J. Genet..

[26] Yoo S., Jin C., Jung D., Choi Y., Choi J., Lee W., Lee S., Lee J., Cha S., Kim C. (2015). Putative functional variants of XRCC1 identified by RegulomeDB were not associated with lung cancer risk in a Korean population. Cancer Genet..

[27] Deyneko I.V., Kalybaeva Y., Kel A.E., Blocker H. (2010). Human-chimpanzee promoter comparisons: Property-conserved evolution?. Genomics.

[28] Boyle A., Hong E., Hariharan M., Cheng Y., Schaub M., Kasowski M., Karczewski K., Park J., Hitz B., Weng S. (2012). Annotation of functional variation in personal genomes using RegulomeDB. Genome Res..

[29] Mathelier A., Fornes O., Arenillas D., Chen C., Denay G., Lee J., Shi W., Shyr C., Tan G., Worsley-Hunt R. (2016). JASPAR 2016: A major expansion and update of the open-access database of transcription factor binding profiles. Nucleic Acids Res..

[30] Yevshin I., Sharipov R., Valeev T., Kel A., Kolpakov F. (2017). GTRD: A database of transcription factor binding sites identified by ChIP-seq experiments. Nucleic Acids Res..

[31] Kulakovskiy I., Vorontsov I., Yevshin I., Sharipov R., Fedorova A., Rumynskiy E., Medvedeva Y., Magana-Mora A., Bajic V., Papatsenko D. (2018). HOCOMOCO: Towards a complete collection of transcription factor binding models for human and mouse via large-scale ChIP-Seq analysis. Nucleic Acids Res..

[32] Levitsky V., Zemlyanskaya E., Oshchepkov D., Podkolodnaya O., Ignatieva E., Grosse I., Mironova V., Merkulova T. (2019). A single ChIP-seq dataset is sufficient for comprehensive analysis of motifs co-occurrence with MCOT package. Nucleic Acids Res..

[33] Sokolenko A., Sandomirskii I., Savinkova L. (1996). Interaction of yeast TATA-binding protein with short promotor segments. Mol. Biol..

[34] Ponomarenko M., Ponomarenko J., Frolov A., Podkolodny N., Savinkova L., Kolchanov N., Overton G. (1999). Identification of sequence-dependent features correlating to activity of DNA sites interacting with proteins. Bioinformatics.

[35] Savinkova L., Drachkova I., Ponomarenko M., Lysova M., Arshinova T., Kolchanov N. (2007). Interaction of recombinant TATA-binding protein with TATA-boxes of mammalian gene promoters. Ecol. Genet..

[36] Ponomarenko P., Savinkova L., Drachkova I., Lysova M., Arshinova T., Ponomarenko M., Kolchanov N. (2008). A step-by-step model of TBP/TATA box binding allows predicting human hereditary diseases by single nucleotide polymorphism. Dokl. Biochem. Biophys..

[37] Delgadillo R., Whittington J., Parkhurst L., Parkhurst L. (2009). The TBP core domain in solution variably bends TATA sequences via a three-step binding mechanism. Biochemistry.

[38] Lu Z. (2011). PubMed and beyond: A survey of web tools for searching biomedical literature. Database.

[39] Ponomarenko P., Suslov V., Savinkova L., Ponomarenko M., Kolchanov N. (2010). A precise equilibrium equation for four steps of binding between TBP and TATA-box allows for the prediction of phenotypical expression upon mutation. Biofizika.

[40] Savinkova L., Drachkova I., Arshinova T., Ponomarenko P., Ponomarenko M., Kolchanov N. (2013). An experimental verification of the predicted effects of promoter TATA-box polymorphisms associated with human diseases on interactions between the TATA boxes and TATA-binding protein. PLoS ONE.

[41] Drachkova I., Savinkova L., Arshinova T., Ponomarenko M., Peltek S., Kolchanov N. (2014). The mechanism by which TATA-box polymorphisms associated with human hereditary diseases influence interactions with the TATA-binding protein. Hum. Mutat..

[42] Drachkova I., Shekhovtsov S., Peltek S., Ponomarenko P., Arshinova T., Ponomarenko M., Merkulova T., Savinkova L., Kolchanov N. (2012). Study of interaction of human TATA-binding protein with TATA-element of NOS2A gene promoter using surface plasmon resonance method. Vavilov. J. Genet. Breed..

[43] Arkova O., Kuznetsov N., Fedorova O., Kolchanov N., Savinkova L. (2014). Realtime interaction between TBP and the TATA box of the human triosephosphate isomerase gene promoter in the norm and pathology. Acta Nat..

[44] Arkova O., Kuznetsov N., Fedorova O., Savinkova L. (2017). A real-time study of the interaction of TBP with a TATA box-containing duplex identical to an ancestral or minor allele of human gene LEP or TPI. J. Biomol. Struct. Dyn..

[45] Ponomarenko M., Rasskazov D., Arkova O., Ponomarenko P., Suslov V., Savinkova L., Kolchanov N. (2015). How to use SNP_TATA_Comparator to find a significant change in gene expression caused by the regulatory SNP of this gene’s promoter via a change in affinity of the TATA-binding protein for this promoter. Biomed. Res. Int..

[46] Ponomarenko M., Rasskazov D., Chadaeva I., Sharypova E., Ponomarenko P., Arkova O., Kashina E., Ivanisenko N., Zhechev D., Savinkova L. (2017). SNP_TATA_Comparator: Genomewide landmarks for preventive personalized medicine. Front. Biosci..

[47] Turnaev I., Rasskazov D., Arkova O., Ponomarenko M., Ponomarenko P., Savinkova L., Kolchanov N. (2016). Hypothetical SNP markers that significantly affect the affinity of the TATA-binding protein to *VEGFA*, *ERBB2*, *IGF1R*, *FLT1*, *KDR*, and *MET* oncogene promoters as chemotherapy targets. Mol. Biol..

[48] Arkova O., Ponomarenko M., Rasskazov D., Drachkova I., Arshinova T., Ponomarenko P., Savinkova L., Kolchanov N. (2015). Obesity-related known and candidate SNP markers can significantly change affinity of TATA-binding protein for human gene promoters. BMC Genom..

[49] Ponomarenko M., Arkova O., Rasskazov D., Ponomarenko P., Savinkova L., Kolchanov N. (2016). Candidate SNP markers of gender-biased autoimmune complications of monogenic diseases are predicted by a significant change in the affinity of TATA-binding protein for human gene promoters. Front. Immunol..

[50] Ponomarenko P., Chadaeva I., Rasskazov D., Sharypova E., Kashina E., Drachkova I., Zhechev D., Ponomarenko M., Savinkova L., Kolchanov N. (2017). Candidate SNP markers of familial and sporadic Alzheimer’s diseases are predicted by a significant change in the affinity of TATA-binding protein for human gene promoters. Front. Aging Neurosci..

[51] Ponomarenko P., Rasskazov D., Suslov V., Sharypova E., Savinkova L., Podkolodnaya O., Podkolodny N., Tverdokhleb N., Chadaeva I., Ponomarenko M. (2016). Candidate SNP markers of chronopathologies are predicted by a significant change in the affinity of TATA-binding protein for human gene promoters. BioMed Res. Int..

[52] Chadaeva I., Ponomarenko M., Rasskazov D., Sharypova E., Kashina E., Matveeva M., Arshinova T., Ponomarenko P., Arkova O., Bondar N. (2016). Candidate SNP markers of aggressiveness-related complications and comorbidities of genetic diseases are predicted by a significant change in the affinity of TATA-binding protein for human gene promoters. BMC Genom..

[53] Chadaeva I., Ponomarenko P., Rasskazov D., Sharypova E., Kashina E., Zhechev D., Drachkova I., Arkova O., Savinkova L., Ponomarenko M. (2018). Candidate SNP markers of reproductive potential are predicted by a significant change in the affinity of TATA-binding protein for human gene promoters. BMC Genom..

[54] Chadaeva I., Ponomarenko P., Rasskazov D., Sharypova E., Kashina E., Kleshchev M., Ponomarenko M., Naumenko V., Savinkova L., Kolchanov N. (2019). Natural selection equally supports the human tendencies in subordination and domination: A genome-wide study with in silico confirmation and in vivo validation in mice. Front. Genet..

[55] Ponomarenko M., Rasskazov D., Chadaeva I., Sharypova E., Drachkova I., Ponomarenko P., Oshchepkova E., Savinkova L., Kolchanov N. (2019). Candidate SNP-markers of atherosclerosis, which may significantly change the affinity of the TATA-binding protein for the human gene promoters. Russ. J. Genet..

[56] Vasileiou P., Mourouzis I., Pantos C. (2017). Principal aspects regarding the maintenance of mammalian mitochondrial genome integrity. Int. J. Mol. Sci..

[57] Koroleva I., Nazarenko M., Kucher A. (2017). Role of microRNA in development of instability of atherosclerotic plaque. Biochemistry.

[58] Plengpanich W., Le Goff W., Poolsuk S., Julia Z., Guerin M., Khovidhunkit W. (2011). CETP deficiency due to a novel mutation in the CETP gene promoter and its effect on cholesterol efflux and selective uptake into hepatocytes. Atherosclerosis.

[59] Waardenberg A., Basset S., Bouveret R., Harvey R. (2015). CompGO: An R package for comparing and visualizing Gene Ontology enrichment differences between DNA binding experiments. BMC Bioinform..

[60] Chen T., Sun M., Wang J., Cui J., Liu Z., Yu B. (2017). A novel swine model for evaluation of dyslipidemia and atherosclerosis induced by human CETP overexpression. Lipids Health Dis..

[61] Casquero A., Berti J., Teixeira L., de Oliveira H. (2017). Chronic exercise reduces CETP and mesterolone treatment counteracts exercise benefits on plasma lipoproteins profile: Studies in transgenic mice. Lipids.

[62] Kasowski M., Grubert F., Heffelfinger C., Hariharan M., Asabere A., Waszak S., Habegger L., Rozowsky J., Shi M., Urban A. (2010). Variation in transcription factor binding among humans. Science.

[63] Abecasis G., Auton A., Brooks L., DePristo M., Durbin R., Handsaker R., Kang H., Marth G., McVean G., 1000 Genomes Project Consortium (2012). An integrated map of genetic variation from 1.092 human genomes. Nature.

[64] Cervera A., Planas A.M., Justicia C., Urra X., Jensenius J., Torres F., Lozano F., Chamorro A. (2010). Genetically-defined deficiency of mannose-binding lectin is associated with protection after experimental stroke in mice and outcome in human stroke. PLoS ONE.

[65] Sziller I., Babula O., Hupuczi P., Nagy B., Rigo B., Szabo G., Papp Z., Linhares I., Witkin S. (2007). Mannose-binding lectin (MBL) codon 54 gene polymorphism protects against development of pre-eclampsia, HELLP syndrome and pre-eclampsia-associated intrauterine growth restriction. Mol. Hum. Reprod..

[66] Boldt A., Culpi L., Tsuneto L., de Souza I., Kun J., Petzl-Erler M. (2006). Diversity of the MBL2 gene in various Brazilian populations and the case of selection at the mannose-binding lectin locus. Hum. Immunol..

[67] Losin I., Shakhnovich R., Zykov K., Ruda M. (2014). Cardiovascular diseases and mannose-binding lectin. Kardiologiia.

[68] Troelsen L., Garred P., Christiansen B., Torp-Pedersen C., Christensen I., Narvestad E., Jacobsen S. (2010). Double role of mannose-binding lectin in relation to carotid intima-media thickness in patients with rheumatoid arthritis. Mol. Immunol..

[69] Matthijsen R., de Winther M., Kuipers D., van der Made I., Weber C., Herias M., Gijbels M., Buurman W. (2009). Macrophage-specific expression of mannose-binding lectin controls atherosclerosis in low-density lipoprotein receptor-deficient mice. Circulation.

[70] Arnaud E., Barbalat V., Nicaud V., Cambien F., Evans A., Morrison C., Arveiler D., Luc G., Ruidavets J., Emmerich J. (2000). Polymorphisms in the 5′ regulatory region of the tissue factor gene and the risk of myocardial infarction and venous thromboembolism: The ECTIM and PATHROS studies. Etude Cas-Temoins de l’Infarctus du Myocarde. Paris Thrombosis case-control Study. Arterioscler. Thromb. Vasc. Biol..

[71] Hasenstab D., Lea H., Hart C., Lok S., Clowes A. (2000). Tissue factor overexpression in rat arterial neointima models thrombosis and progression of advanced atherosclerosis. Circulation.

[72] Lin H., Yen H., Hsieh S., An L., Shen K. (2014). Low-dose aspirin ameliorated hyperlipidemia, adhesion molecule, and chemokine production induced by high-fat diet in Sprague-Dawley rats. Drug Dev. Res..

[73] Ebert J., Wilgenbus P., Teiber J., Jurk K., Schwierczek K., Dohrmann M., Xia N., Li H., Spiecker L., Ruf W. (2018). Paraoxonase-2 regulates coagulation activation through endothelial tissue factor. Blood.

[74] Watanabe M., Zingg B., Mohrenweiser H. (1996). Molecular analysis of a series of alleles in humans with reduced activity at the triosephosphate isomerase locus. Am. J. Hum. Genet..

[75] Vives-Corrons J., Rubinson-Skala H., Mateo M., Estella J., Feliu E., Dreyfus J. (1978). Triosephosphate isomerase deficiency with hemolytic anemia and severe neuromuscular disease: Familial and biochemical studies of a case found in Spain. Hum. Genet..

[76] Balla G., Vercellotti G., Eaton J., Jacob H. (1990). Heme uptake by endothelium synergizes polymorphonuclear granulocyte-mediated damage. Trans. Assoc. Am. Physicians.

[77] Kioumourtzoglou M., Seals R., Gredal O., Mittleman M., Hansen J., Weisskopf M. (2016). Cardiovascular disease and diagnosis of amyotrophic lateral sclerosis: A population based study. Amyotroph. Lateral. Scler. Frontotemporal. Degener..

[78] Martiney J., Cerami A., Slater A. (1996). Inhibition of hemozoin formation in Plasmodium falciparum trophozoite extracts by heme analogs: Possible implication in the resistance to malaria conferred by the beta-thalassemia trait. Mol. Med..

[79] Wang H., Luo W., Wang J., Guo C., Wolffe S., Wang J., Sun E., Bradley K., Campbell A., Eitzman D. (2013). Paradoxical protection from atherosclerosis and thrombosis in a mouse model of sickle cell disease. Br. J. Haematol..

[80] Collins F., Weissman S. (1984). The molecular genetics of human hemoglobin. Prog. Nucleic Acid Res. Mol. Biol..

[81] Gall T., Petho D., Nagy A., Hendrik Z., Mehes G., Potor L., Gram M., Åkerstrom B., Smith A., Nagy P. (2018). Heme induces endoplasmic reticulum stress (HIER stress) in human aortic smooth muscle cells. Front. Physiol..

[82] Nalls M., Wilson J., Patterson N., Tandon A., Zmuda J., Huntsman S., Garcia M., Hu D., Li R., Beamer B. (2008). Admixture mapping of white cell count: Genetic locus responsible for lower white blood cell count in the Health ABC and Jackson Heart studies. Am. J. Hum. Genet..

[83] Michon P., Woolley I., Wood E.M., Kastens W., Zimmerman P., Adams J. (2001). Duffy-null promoter heterozygosity reduces DARC expression and abrogates adhesion of the *P. vivax* ligand required for blood-stage infection. FEBS Lett..

[84] Wan W., Liu Q., Lionakis M., Marino A., Anderson S., Swamydas M., Murphy P. (2015). Atypical chemokine receptor 1 deficiency reduces atherogenesis in ApoE-knockout mice. Cardiovasc. Res..

[85] Gencer S., van der Vorst E.P.C., Aslani M., Weber C., Doring Y., Duchene J. (2019). Atypical chemokine receptors in cardiovascular disease. Thromb. Haemost..

[86] Hobbs M., Udhayakumar V., Levesque M., Booth J., Roberts J., Tkachuk A., Pole A., Coon H., Kariuki S., Nahlen B. (2002). A new NOS2 promoter polymorphism associated with increased nitric oxide production and protection from severe malaria in Tanzanian and Kenyan children. Lancet.

[87] Clark I., Rockett K., Burgner D. (2003). Genes, nitric oxide and malaria in African children. Trends Parasitol..

[88] Zhao J., Shyue S., Lin S., Wei J., Lee T. (2014). Excess nitric oxide impairs LXR(α)-ABCA1-dependent cholesterol efflux in macrophage foam cells. J. Cell Physiol..

[89] Jovanovic A., Sudar-Milovanovic E., Obradovic M., Pitt S., Stewart A., Zafirovic S., Stanimirovic J., Radak D., Isenovic E. (2017). Influence of a high-fat diet on cardiac iNOS in female rats. Curr. Vasc. Pharmacol..

[90] Ponnuswamy P., Ostermeier E., Schrottle A., Chen J., Huang P., Ertl G., Nieswandt B., Kuhlencordt P. (2009). Oxidative stress and compartment of gene expression determine proatherosclerotic effects of inducible nitric oxide synthase. Am. J. Pathol..

[91] Kavlie A., Hiltunen L., Rasi V., Prydz H. (2003). Two novel mutations in the human coagulation factor VII promoter. Thromb. Haemost..

[92] Zacharski L., Delprete S., Kisiel W., Hunt J., Cornell C., Marrin C. (1988). Atherosclerosis and coronary bypass surgery in hereditary factor VII deficiency. Am. J. Med..

[93] Roche H., Zampelas A., Knapper J., Webb D., Brooks C., Jackson K., Wright J., Gould B., Kafatos A., Gibney M. (1998). Effect of long-term olive oil dietary intervention on postprandial triacylglycerol and factor VII metabolism. Am. J. Clin. Nutr..

[94] Mitropoulos K., Esnouf M., Meade T. (1987). Increased factor VII coagulant activity in the rabbit following diet-induced hypercholesterolaemia. Evidence for increased conversion of VII to alpha VIIa and higher flux within the coagulation pathway. Atherosclerosis.

[95] Setorki M., Rafieian-Kopaei M., Merikhi A., Heidarian E., Shahinfard N., Ansari R., Nasri H., Esmael N., Baradaran A. (2013). Suppressive impact of anethum graveolens consumption on biochemical risk factors of atherosclerosis in hypercholesterolemic rabbits. Int. J. Prev. Med..

[96] Niemann S., Broom W., Brown R.H. (2007). Analysis of a genetic defect in the TATA box of the SOD1 gene in a patient with familial amyotrophic lateral sclerosis. Muscle Nerve.

[97] Turner M., Goldacre R., Ramagopalan S., Talbot K., Goldacre M. (2013). Autoimmune disease preceding amyotrophic lateral sclerosis: An epidemiologic study. Neurology.

[98] Ende N., Weinstein F., Chen R., Ende M. (2000). Human umbilical cord blood effect on sod mice (amyotrophic lateral sclerosis). Life Sci..

[99] Charles B., Hsieh M., Adeyemo A., Shriner D., Ramos E., Chin K., Srivastava K., Zakai N., Cushman M., McClure L. (2018). Analyses of genome wide association data, cytokines, and gene expression in African-Americans with benign ethnic neutropenia. PLoS ONE.

[100] Simmons G., Padilla J., Jenkins N., Laughlin M. (2014). Exercise training and vascular cell phenotype in a swine model of familial hypercholesterolaemia: Conduit arteries and veins. Exp. Physiol..

[101] Li W., Tang C., Jin H., Du J. (2011). Effects of onion extract on endogenous vascular H2S and adrenomedulin in rat atherosclerosis. Curr. Pharm. Biotechnol..

[102] Yang H., Zhou L., Wang Z., Roberts L.J., Lin X., Zhao Y., Guo Z. (2009). Overexpression of antioxidant enzymes in ApoE-deficient mice suppresses benzo(a)pyrene-accelerated atherosclerosis. Atherosclerosis.

[103] Cao W., Ning J., Yang X., Liu Z. (2011). Excess exposure to insulin is the primary cause of insulin resistance and its associated atherosclerosis. Curr. Mol. Pharmacol..

[104] Liu Z., Zhou Z., Huang G., Xiao Y., Li Z., Liu C., Na R. (2016). Long-term effects intensive medical therapy on the development and progression of subclinical atherosclerosis and the metabolic syndrome in Chinese patients with type 2 diabetes mellitus. Medicine.

[105] Badin J., Kole A., Stivers B., Progar V., Pareddy A., Alloosh M., Sturek M. (2018). Alloxan-induced diabetes exacerbates coronary atherosclerosis and calcification in Ossabaw miniature swine with metabolic syndrome. J. Transl. Med..

[106] Ferguson J., Ryan M., Gibney E., Brennan L., Roche H., Reilly M. (2014). Dietary isoflavone intake is associated with evoked responses to inflammatory cardiometabolic stimuli and improved glucose homeostasis in healthy volunteers. Nutr. Metab. Cardiovasc. Dis..

[107] Starodubtseva N., Sobolev V., Soboleva A., Nikolaev A., Bruskin S. (2011). Genes expression of metalloproteinases (MMP-1, MMP-2, MMP-9, and MMP-12) associated with psoriasis. Russ. J. Genet..

[108] Manetti M., Ibba-Manneschi L., Fatini C., Guiducci S., Cuomo G., Bonino C., Bazzichi L., Liakouli V., Giacomelli R., Abbate R. (2010). Association of a functional polymorphism in the matrix metalloproteinase-12 promoter region with systemic sclerosis in an Italian population. J. Rheumatol..

[109] Hunninghake G., Cho M., Tesfaigzi Y., Soto-Quiros M., Avila L., Lasky-Su J., Stidley C., Melen E., Soderhall C., Hallberg J. (2009). MMP12, lung function, and COPD in high-risk populations. N. Engl. J. Med..

[110] Hemminki K., Li X., Sundquist J., Sundquist K. (2010). Subsequent autoimmune or related disease in asthma patients: Clustering of diseases or medical care?. Ann. Epidemiol..

[111] Motterle A., Xiao Q., Kiechl S., Pender S., Morris G., Willeit J., Caulfield M., Ye S. (2012). Influence of matrix metalloproteinase-12 on fibrinogen level. Atherosclerosis.

[112] Bukowska H., Pieczul-Mroz J., Jastrzebska M., Chelstowski K., Naruszewicz M. (1998). Decrease in fibrinogen and LDL-cholesterol levels upon supplementation of diet with Lactobacillus plantarum in subjects with moderately elevated cholesterol. Atherosclerosis.

[113] Liang J., Liu E., Yu Y., Kitajima S., Koike T., Jin Y., Morimoto M., Hatakeyama K., Asada Y., Watanabe T. (2006). Macrophage metalloelastase accelerates the progression of atherosclerosis in transgenic rabbits. Circulation.

[114] Liu S., Bajpai A., Hawthorne E., Bae Y., Castagnino P., Monslow J., Pure E., Spiller K., Assoian R. (2019). Cardiovascular protection in females linked to estrogen-dependent inhibition of arterial stiffening and macrophage MMP12. JCI Insight.

[115] Yoshikawa H., Kawai K., Inoue S., Murayama Y., Fujieda K., Kuzuya N., Fujita T., Koide Y., Yamashita K. (1989). Hyperglucagonemia of insulin autoimmune syndrome induced by methimazole in a patient with Graves’ disease. Endocrinol. Jpn..

[116] White J., Saunders G. (1986). Structure of the human glucagon gene. Nucleic Acids Res..

[117] Hubbard R., Kosch C., Sanchez A., Sabate J., Berk L., Shavlik G. (1989). Effect of dietary protein on serum insulin and glucagon levels in hyper- and normocholesterolemic men. Atherosclerosis.

[118] Galassetti P., Larson J., Iwanaga K., Salsberg S., Eliakim A., Pontello A. (2006). Effect of a high-fat meal on the growth hormone response to exercise in children. J. Pediatr. Endocrinol. Metab..

[119] Zhang Y., Proenca R., Maffei M., Barone M., Leopold L., Friedman J.M. (1994). Positional cloning of the mouse obese gene and its human homologue. Nature.

[120] Skrypnik K., Suliburska J., Skrypnik D., Pilarski L., Regula J., Bogdanski P. (2017). The genetic basis of obesity complications. Acta Sci. Pol. Technol. Aliment..

[121] Jun J., Ma Z., Pyla R., Segar L. (2012). Leptin treatment inhibits the progression of atherosclerosis by attenuating hypercholesterolemia in type 1 diabetic Ins2(+/Akita):apoE(-/-) mice. Atherosclerosis.

[122] Matsunaga A., Sasaki J., Han H., Huang W., Kugi M., Koga T., Ichiki S., Shinkawa T., Arakawa K. (1999). Compound heterozygosity for an apolipoprotein A1 gene promoter mutation and a structural nonsense mutation with apolipoprotein A1 deficiency. Arterioscler. Thromb. Vasc. Biol..

[123] Reimers G., Jackson C., Rickards J., Chan P., Cohn J., Rye K., Barter P., Rodgers K. (2011). Inhibition of rupture of established atherosclerotic plaques by treatment with apolipoprotein A-I. Cardiovasc. Res..

[124] Vermeulen A. (1990). Plasma lipid and lipoprotein levels in obese post-menopausal women: Effects of a short-term low-protein diet and exercise. Maturitas.

[125] Priyadarshini S., Pradhan B., Griebel P., Aich P. (2018). Cortisol regulates immune and metabolic processes in murine adipocytes and macrophages through HTR2c and HTR5a serotonin receptors. Eur. J. Cell Biol..

[126] Lord C., Wyler S., Wan R., Castorena C., Ahmed N., Mathew D., Lee S., Liu C., Elmquist J. (2017). The atypical antipsychotic olanzapine causes weight gain by targeting serotonin receptor 2C. J. Clin. Investig..

[127] Strandberg L., Mellstrom D., Ljunggren O., Grundberg E., Karlsson M., Holmberg A., Orwoll E., Eriksson A., Svedberg J., Bengtsson M. (2008). IL6 and IL1B polymorphisms are associated with fat mass in older men: The MrOS Study Sweden. Obesity.

[128] Hayashi F., Watanabe M., Nanba T., Inoue N., Akamizu T., Iwatani Y. (2009). Association of the -31C/T functional polymorphism in the interleukin-1beta gene with the intractability of Graves’ disease and the proportion of T helper type 17 cells. Clin. Exp. Immunol..

[129] Borkowska P., Kucia K., Rzezniczek S., Paul-Samojedny M., Kowalczyk M., Owczarek A., Suchanek R., Medrala T., Kowalski J. (2011). Interleukin-1beta promoter (-31T/C and -511C/T) polymorphisms in major recurrent depression. J. Mol. Neurosci..

[130] Wu K., Zhou X., Zheng F., Xu X., Lin Y., Yang J. (2010). Influence of interleukin-1 beta genetic polymorphism, smoking and alcohol drinking on the risk of non-small cell lung cancer. Clin. Chim. Acta.

[131] Wang Y., Kato N., Hoshida Y., Yoshida H., Taniguchi H., Goto T., Moriyama M., Otsuka M., Shiina S., Shiratori Y. (2003). Interleukin-1beta gene polymorphisms associated with hepatocellular carcinoma in hepatitis C virus infection. Hepatology.

[132] El-Omar E., Carrington M., Chow W., McColl K., Bream J., Young H., Herrera J., Lissowska J., Yuan C., Rothman N. (2000). Interleukin-1 polymorphisms associated with increased risk of gastric cancer. Nature.

[133] Martinez-Carrillo D., Garza-Gonzalez E., Betancourt-Linares R., Monico-Manzano T., Antunez-Rivera C., Roman-Roman A., Flores-Alfaro E., Illades-Aguiar B., Fernandez-Tilapa G. (2010). Association of IL1B -511C/-31T haplotype and Helicobacter pylori vacA genotypes with gastric ulcer and chronic gastritis. BMC Gastroenterol..

[134] Cochain C., Vafadarnejad E., Arampatzi P., Pelisek J., Winkels H., Ley K., Wolf D., Saliba A., Zernecke A. (2018). Single-cell RNA-seq reveals the transcriptional landscape and heterogeneity of aortic macrophages in murine atherosclerosis. Circ. Res..

[135] Zhou Q., Han X., Li R., Zhao W., Bai B., Yan C., Dong X. (2018). Anti-atherosclerosis of oligomeric proanthocyanidins from Rhodiola rosea on rat model via hypolipemic, antioxidant, anti-inflammatory activities together with regulation of endothelial function. Phytomedicine.

[136] Giunzioni I., Bonomo A., Bishop E., Castiglioni S., Corsini A., Bellosta S. (2014). Cigarette smoke condensate affects monocyte interaction with endothelium. Atherosclerosis.

[137] Abbas A., Lechevrel M., Sichel F. (2006). Identification of new single nucleotid polymorphisms (SNP) in alcohol dehydrogenase class IV ADH7 gene within a French population. Arch. Toxicol..

[138] Lainas P., Fuks D., Gaujoux S., Machroub Z., Fregeville A., Perniceni T., Mal F., Dousset B., Gayet B. (2017). Preoperative imaging and prediction of oesophageal conduit necrosis after oesophagectomy for cancer. Br. J. Surg..

[139] Peltoketo H., Piao Y., Mannermaa A., Ponder B., Isomaa V., Poutanen M., Winqvist R., Vihko R. (1994). A point mutation in the putative TATA box, detected in nondiseased individuals and patients with hereditary breast cancer, decreases promoter activity of the 17 beta-hydroxysteroid dehydrogenase type 1 gene 2 (EDH17B2) in vitro. Genomics.

[140] Ma A., Wang D., An Y., Fang W., Zhu H. (2017). Comparative transcriptomic analysis of mice liver treated with different AMPK activators in a mice model of atherosclerosis. Oncotarget.

[141] He W., Gauri M., Li T., Wang R., Lin S. (2016). Current knowledge of the multifunctional 17β-hydroxysteroid dehydrogenase type 1 (HSD17B1). Gene.

[142] Chistiakov D., Sobenin I., Bobryshev Y., Orekhov A. (2012). Mitochondrial dysfunction and mitochondrial DNA mutations in atherosclerotic complications in diabetes. World J. Cardiol..

[143] Cervelli T., Borghini A., Galli A., Andreassi M. (2012). DNA damage and repair in atherosclerosis: Current insights and future perspectives. Int. J. Mol. Sci..

[144] Coxhead J., Williams E., Mathers J. (2005). DNA mismatch repair status may influence anti-neoplastic effects of butyrate. Biochem. Soc. Trans..

[145] Liu H., Qu X., Yuan F., Zhang M., Fang W. (2013). A RTK-based functional RNAi screen reveals determinants of PTX-3 expression. Int. J. Clin. Exp. Pathol..

[146] Philips S., Richter A., Oesterreich S., Rae J., Flockhart D., Perumal N., Skaar T. (2012). Functional characterization of a genetic polymorphism in the promoter of the ESR2 gene. Horm. Cancer.

[147] McRobb L.S., McGrath K.C.Y., Tsatralis T., Liong E.C., Tan J.T.M., Hughes G., Handelsman D.J., Heather A.K. (2017). Estrogen receptor control of atherosclerotic calcification and smooth muscle cell osteogenic differentiation. Arterioscler. Thromb.Vasc. Biol..

[148] Anderson J.J., Kruszka B., Delaney J.A., He K., Burke G.L., Alonso A., Bild D.E., Budoff M., Michos E.D. (2016). Calcium intake from diet and supplements and the risk of coronary artery calcification and its progression among older adults: 10-year follow-up of the Multi-Ethnic Study of Atherosclerosis (MESA). J. Am. Heart Assoc..

[149] Al-Shakfa F., Dulucq S., Brukner I., Milacic I., Ansari M., Beaulieu P., Moghrabi A., Laverdiere C., Sallan S., Silverman L. (2009). DNA variants in region for noncoding interfering transcript of dihydrofolate reductase gene and outcome in childhood acute lymphoblastic leukemia. Clin. Cancer Res..

[150] Harrison D., Chen W., Dikalov S., Li L. (2010). Regulation of endothelial cell tetrahydrobiopterin pathophysiological and therapeutic implications. Adv. Pharmacol..

[151] Okumura K., Tsukamoto H. (2011). Folate in smokers. Clin. Chim. Acta.

[152] Wilhelmson A., Bourghardt-Fagman J., Gogos J., Fogelstrand P., Tivesten A. (2011). Catechol-O-methyltransferase is dispensable for vascular protection by estradiol in mouse models of atherosclerosis and neointima formation. Endocrinology.

[153] Lievens D., Habets K., Robertson A., Laouar Y., Winkels H., Rademakers T., Beckers L., Wijnands E., Boon L., Mosaheb M. (2013). Abrogated transforming growth factor beta receptor II (TGFβRII) signalling in dendritic cells promotes immune reactivity of T cells resulting in enhanced atherosclerosis. Eur. Heart J..

[154] Lou J., Zhao D., Zhang L., Song S., Li Y., Sun F., Ding X., Yu C., Li Y., Liu M. (2016). Type III transforming growth factor-β receptor drives cardiac hypertrophy through β-arrestin2-dependent activation of calmodulin-dependent protein kinase II. Hypertension.

[155] Dol-Gleizes F., Delesque-Touchard N., Mares A., Nestor A., Schaeffer P., Bono F. (2013). A new synthetic FGF receptor antagonist inhibits arteriosclerosis in a mouse vein graft model and atherosclerosis in apolipoprotein E-deficient mice. PLoS ONE.

[156] Yu D., Rui X., He S. (2014). Effect of heparin-derived oligosaccharide on bFGFR1 and bFGFR2 in vascular smooth muscle cells. Vasc. Endovasc. Surg..

[157] Hartgerink J., Cramm J., de Vos A., Bakker T., Steyerberg E., Mackenbach J., Nieboer A. (2014). Situational awareness, relational coordination and integrated care delivery to hospitalized elderly in the Netherlands: A comparison between hospitals. BMC Geriatr..

[158] Yu E., Calvert P., Mercer J., Harrison J., Baker L., Figg N., Kumar S., Wang J., Hurst L., Obaid D. (2013). Mitochondrial DNA damage can promote atherosclerosis independently of reactive oxygen species through effects on smooth muscle cells and monocytes and correlates with higher-risk plaques in humans. Circulation.

[159] Hu Q., Ren J., Li G., Wu J., Wu X., Wang G., Gu G., Ren H., Hong Z., Li J. (2018). The mitochondrially targeted antioxidant MitoQ protects the intestinal barrier by ameliorating mitochondrial DNA damage via the Nrf2/ARE signaling pathway. Cell Death Dis..

[160] Cioffi F., Senese R., Lasala P., Ziello A., Mazzoli A., Crescenzo R., Liverini G., Lanni A., Goglia F., Iossa S. (2017). Fructose-rich diet affects mitochondrial DNA damage and repair in rats. Nutrients.

[161] Reddyvari H., Govatati S., Matha S., Korla S., Malempati S., Pasupuleti S., Bhanoori M., Nallanchakravarthula V. (2017). Therapeutic effect of green tea extract on alcohol induced hepatic mitochondrial DNA damage in albino wistar rats. J. Adv. Res..

[162] Stein S., Lohmann C., Handschin C., Stenfeldt E., Boren J., Luscher T., Matter C. (2010). ApoE-/- PGC-1α-/- mice display reduced IL-18 levels and do not develop enhanced atherosclerosis. PLoS ONE.

[163] Zhao Q., Zhang J., Wang H. (2015). PGC-1α limits angiotensin II-induced rat vascular smooth muscle cells proliferation via attenuating NOX1-mediated generation of reactive oxygen species. Biosci. Rep..

[164] Vernochet C., Mourier A., Bezy O., Macotela Y., Boucher J., Rardin M., An D., Lee K., Ilkayeva O., Zingaretti C. (2012). Adipose-specific deletion of TFAM increases mitochondrial oxidation and protects mice against obesity and insulin resistance. Cell Metab..

[165] Yoshida T., Azuma H., Aihara K., Fujimura M., Akaike M., Mitsui T., Matsumoto T. (2005). Vascular smooth muscle cell proliferation is dependent upon upregulation of mitochondrial transcription factor A (mtTFA) expression in injured rat carotid artery. Atherosclerosis.

[166] Han D., Johnson H., Rao M., Martin G., Sancheti H., Silkwood K., Decker C., Nguyen K., Casian J., Cadenas E. (2017). Mitochondrial remodeling in the liver following chronic alcohol feeding to rats. Free Radic. Biol. Med..

[167] Mercer J., Cheng K., Figg N., Gorenne I., Mahmoudi M., Griffin J., Vidal-Puig A., Logan A., Murphy M., Bennett M. (2010). DNA damage links mitochondrial dysfunction to atherosclerosis and the metabolic syndrome. Circ. Res..

[168] Mercer J., Yu E., Figg N., Cheng K., Prime T., Griffin J., Masoodi M., Vidal-Puig A., Murphy M., Bennett M. (2012). The mitochondria-targeted antioxidant MitoQ decreases features of the metabolic syndrome in ATM+/-/ApoE-/- mice. Free Radic. Biol. Med..

[169] Liu L., Nagai I., Gao Y., Matsushima Y., Kawai Y., Sayama K. (2017). Effects of catechins and caffeine on the development of atherosclerosis in mice. Biosci. Biotechnol. Biochem..

[170] Razani B., Feng C., Semenkovich C. (2010). p53 is required for chloroquine-induced atheroprotection but not insulin sensitization. J. Lipid Res..

[171] Nair V.D. (2006). Activation of p53 signaling initiates apoptotic death in a cellular model of Parkinson’s disease. Apoptosis.

[172] Stamatelopoulos K., Karatzi K., Sidossis L. (2009). Noninvasive methods for assessing early markers of atherosclerosis: The role of body composition and nutrition. Curr. Opin. Clin. Nutr. Metab. Care..

[173] Teng I., Tsai M., Shih S., Tsuei B., Chang H., Chuang Y., Lin C., Chern C., Chen S. (2018). Chalcone derivatives enhance ATP-binding cassette transporters A1 in human THP-1 macrophages. Molecules.

[174] Liang H., Wang Y. (2018). Berberine alleviates hepatic lipid accumulation by increasing ABCA1 through the protein kinase C δ pathway. Biochem. Biophys. Res. Commun..

[175] Wang H., Liu D., Zhang H. (2019). Investigation of the underlying genes and mechanism of macrophage-enriched ruptured atherosclerotic plaques using bioinformatics method. J. Atheroscler. Thromb..

[176] Kumar S., Kim C., Simmons R., Jo H. (2014). Role of flow-sensitive microRNAs in endothelial dysfunction and atherosclerosis: Mechanosensitive athero-miRs. Arterioscler. Thromb. Vasc. Biol..

[177] Hergenreider E., Heydt S., Treguer K., Boettger T., Horrevoets A., Zeiher A., Scheffer M., Frangakis A., Yin X., Mayr M. (2012). Atheroprotective communication between endothelial cells and smooth muscle cells through miRNAs. Nat. Cell Biol..

[178] Castiglioni S., Monti M., Arnaboldi L., Canavesi M., Ainis Buscherini G., Calabresi L., Corsini A., Bellosta S. (2017). ABCA1 and HDL3 are required to modulate smooth muscle cells phenotypic switch after cholesterol loading. Atherosclerosis.

[179] Zhang H., Wang Y., Cao C., Yang X., Ma S., Han X., Yang X., Yang A., Tian J., Xu H. (2016). A regulatory circuit involving miR-143 and DNMT3a mediates vascular smooth muscle cell proliferation induced by homocysteine. Mol. Med. Rep..

[180] Liu K., Xuekelati S., Zhang Y., Yin Y., Li Y., Chai R., Li X., Peng Y., Wu J., Guo X. (2017). Expression levels of atherosclerosis-associated miR-143 and miR-145 in the plasma of patients with hyperhomocysteinaemia. BMC Cardiovasc. Disord..

[181] Hahn S., Buratowski S., Sharp P., Guarente L. (1989). Yeast TATA-binding protein TFIID binds to TATA elements with both consensus and nonconsensus DNA sequences. Proc. Natl. Acad. Sci. USA.

[182] Bucher P. (1990). Weight matrix descriptions of four eukaryotic RNA polymerase II promoter elements derived from 502 unrelated promoter sequences. J. Mol. Biol..

[183] Karas H., Knuppel R., Schulz W., Sklenar H., Wingender E. (1996). Combining structural analysis of DNA with search routines for the detection of transcription regulatory elements. Comput. Applic. Biosci..

[184] Pugh B. (1995). Purification of the human TATA-binding protein, TBP. Methods Mol. Biol..

