# Supplementary material for: Candidate SNP Markers of Atherogenesis Significantly Shifting the Affinity of TATA-Binding Protein for Human Gene Promoters Show Stabilizing Natural Selection as a Sum of Neutral Drift Accelerating Atherogenesis and Directional Natural Selection Slowing It
Source: Int J Mol Sci. 2020 Feb 5;21(3):1045. doi: 10.3390/ijms21031045 (PMC7037642; doi:10.3390/ijms21031045)
Supplement: Supplementary file 1 [file ijms-21-01045-s001.zip › Ponomarenko_!_Int_J_Mol_Sci_Supplementary-Materials_R0.pdf]

International Journal of Molecular Sciences

MDPI

SUPPLEMENTARY MATERIALS

Candidate SNP markers for atherogenesis significantly shifting the affinity of TATA-binding protein for human gene promoters show stabilizing natural selection as sum of neutral drift accelerating atherogenesis and directional natural selection slowing it

Mikhail Ponomarenko[1,2*], Dmitry Rasskazov[1], Irina Chadaeva[1,2], Ekaterina Sharypova[1], Irina Drachkova[1], Dmitry Oshchepkov[1], Petr Ponomarenko[1], Ludmila Savinkova[1], Eugene Oshchepkova[1], Maria Nazarenko[3], Nikolay Kolchanov[1,2]

[1] *Institute of Cytology and Genetics, Siberian Branch of Russian Academy of Sciences, Novosibirsk 630090, Russia; pon@bionet.nsc.ru;*

[2] *Novosibirsk State University, Novosibirsk 630090, Russia; ichadaeva@bionet.nsc.ru;*

[3] *Institute of Medical Genetics, Tomsk National Research Medical Center, Russian Academy of Science, Tomsk, 634009, Russia; maria.nazarenko@medgenetics.ru;*

[#] *Correspondence: pon@bionet.nsc.ru. Tel.: +7 (383) 363-49-63 ext. 1311 (M.P.)*

S1. Supplementary DNA sequence analysis

Two DNA sequence $S_{WT}=\{s_{WT;-90}\ldots s_{WT;i}\ldots s_{WT;-1}\}$ and $S_{SNP}=\{s_{SNP;-90}\ldots s_{SNP;i}\ldots s_{SNP;-1}\}$, which lengths are 90 bp that correspond to two variants of a given promoter located immediately upstream of the transcription start site (TSS, $s_{1;0}=s_{2;0}$; $s_i \in \{a, c, g, t\}$) are the input data of our earlier created Web service SNP_TATA_Comparator (http://beehive.bionet.nsc.ru/cgi-bin/mgs/tatascan_fox/start.pl) [45] used, as shown in Figure 1c (hereinafter, see the main section "2. Results and Discussion").

First of all, the affinity estimate "-ln(K$_D$(S))" is calculated upon each of these sequences $S \in \{S_{WT}, S_{SNP}\}$, as:

$$-\ln(K_D) = 10.9 - 0.2\,\{\ln(K_{SLIDE}) + \ln(K_{STOP}) + \ln(K_{BEND})\}, \quad (1)$$

where 10.9 (ln units) and 0.2 corresponds to the estimates of nonspecific TBP-DNA affinity (i.e., 10 mM [181] and the stoichiometric coefficient [36]; K_{STOP} as an empirical estimate of an impact of the TBP stops at the most probable TBP-binding site according to Bucher's rule [182] namely:

$$\boldsymbol{ln(K_{STOP})} = \underset{(+),(-)\ \boldsymbol{DNA\ chains}}{\mathbf{MAX}} \left\{\textstyle\sum_{j=-1}^{13} \boldsymbol{w_{j;s_{i+j}}}\right\}; \quad (2)$$

where w_{js} as an element of the Bucher's position-weight matrix [182], which corresponds to the case of the nucleotide s located within j-th position of the DNP sequence analyzed.

In Eq. (1), K_{SLIDE} as an empirical estimate of an impact of the TBP sliding along DNA near the most probable TBP-binding site mentioned above (i.e., DNA sequence region [TBP-DNA contact ± 5bp]) is heuristically calculated, as:

$$-\ln(K_{SLIDE}) = \mathbf{MEAN}_{[\text{TBP-DNA contact}\pm 5\text{bp}]}\,\{0.8[TA] + 3.4\mu + 35.1\}, \quad (3)$$

where [TA] as as weighted number of dinucleotide TA; μ as the arithmetical mean of the minor groove width of the DNA helix [183] of the TBP-binding site under consideration; 0.8, 3.4, and 35.1 as regression coefficients [34]

In Eq. (1), K_{BEND} as an empirical estimate of an impact of the DNA helix bend stabilizing TBP-DNA complex is calculated, namely:

$$-\ln(K_{BEND}) = \mathbf{MEAN}_{TBP\text{-}DNA}\ \{0.9[TA, AA, TG, AG] + 2.5[TA, TC, TG] + 14.4\}, \quad \mathbf{(4)}$$

where $MEAN_{TBP\text{-}DNA}$ as the arithmetical mean of both DNA strands of the TBP-DNA complex under consideration (see Eq. (2)); 0.9, 2.5, and 14.4 as regression coefficients [34].

Additionally, the "-ln[K_D]" values (Eq. 1) are accompanied by its standard deviation estimates (δ) according to all the possible nucleotide substitutions, $s_{\bullet;j} \rightarrow \xi$, at each position j of the above regions [TBP-DNA contact ±5bp], such as:

$$\delta(S_\bullet)=[(\Sigma_{1\leq i\leq 26}\Sigma_{\xi\circledcirc\{a,c,g,t\}}[\ln(K_D(\{s_{\bullet;i-13}\ldots\xi\ldots\bullet s_{\bullet;i+12}\})/K_D(\{s_{\bullet;i-13}\ldots s_{\bullet;i+j}\ldots s_{\bullet;i+12}\})^2])/(3*26)]^{1/2} \quad \mathbf{(5)}$$

Finally, two estimates "$-\ln(K_D(S_{WT}))\pm\delta(S_{WT})$" and "$-\ln(K_D(S_{SNP}))\pm\delta(S_{SNP})$" calculated upon the input sequences S_{WT} and S_{SNP} (Eqs. (1–5)) were statistically compared with one another in the terms of Fisher's Z-score, such as:

$$Z = \mathbf{abs}[\ln(K_{WT;D}/K_{SNP;D})]/[\delta_{WT}^2+\delta_{SNP;}^2]^{1/2}. \quad (6)$$

where Z as the above Z-score pinpointing *p*-value of the probability estimate of acceptance of the H_0-hypothesis "H_0: $K_D(S_{WT}) \neq K_D(S_{SNP})$", which was taken from the commonly accepted statistical package R [59].

On this basis, the final decision is made at its statistically significant level $\alpha < 0.05$ (where $\alpha = 1\text{-}p$), namely:

IF {*INEQUALITY* "$-\ln(K_{WT;D}) > -\ln(K_{SNP;D})$" is statistically significant},

THEN {*DECISION* is "S_{SNP} provides an underexpression of a given gene in comparison with S_{WT}, which is the norm"};

ELSE [**IF** {*INEQUALITY* "$-\ln(K_{WT;D}) < -\ln(K_{SNP;D})$" is statistically significant},

THEN {*DECISION* is "S_{SNP} provides an overexpression of a given gene in comparison with S_{WT}, which is the norm"},]

OTHERWISE {*DECISION* is "alteration of the expression of this gene is insignificant"}.

One can see this DECISION in Figure 1c, such as: the text box "Result", the line "DECISION".

S2. Supplementary *in vitro* measurements

Recombinant full-length human TBP was expressed in Escherichia coli BL21 (DE3) cells transformed with the pAR3038-TBP plasmid (a kind gift from Prof. B. Pugh, Pennsylvania State University) by a previously described method [184] with two modifications: the IPTG concentration was 1.0 instead of 0.1 mM; the induction time was 3 instead of 1.5 h. For details of our protocol for production and purification of human TBP, see ref. [40].

ODNs 26 bp in length were synthesized by the Biosynthesis Enterprise (Novosibirsk, Russia) and were purified by PAGE. Labeled double-stranded ODNs were prepared by ^{32}P labeling of both strands by means of T4 polynucleotide kinase (SibEnzyme, Novosibirsk) with subsequent annealing by heating to 95°C (at equimolar concentrations) and slow cooling (no less than 3 h) to room temperature. The duplexes were analyzed in a 15% nondenaturing polyacrylamide gel (1 × Tris-borate-EDTA buffer) and isolated by electroelution. For more details of our protocol for labeling of ODNs with ^{32}P, readers can see Ref. [40].

The equilibrium dissociation constants (K_D) were determined for the complexes of TBP with each 26-bp ODN in question. Experiments on association kinetics were conducted at four ODN concentrations (Figure. 3a). The experiments with TBP–ODN binding were carried out at 25°C in binding buffer (20 mM 4-[2-hydroxyethyl]-1-piperazineethanesulfonic acid [HEPES]-KOH pH 7.6, 5 mM MgCl2, 70 mM KCl, 1 mM dithiothreitol (DTT), 100 μg/mL BSA, 0.01% of NP-40, and 5% of glycerol) at a fixed concentration (0.3 nM) of active TBP. The gels were dried, and Imaging Screen-K (Kodak, Rochester, NY, USA) was exposed to these gels for analysis on a Molecular Imager PharosFX Plus phosphorimager (Bio-Rad, Herts, UK). The resulting autoradiographs were quantitated in the Quantity One 4.5.0 software (Bio-Rad) as displayed in Figures 3b. Using these data as input for publicly available software Graph-Pad Prism 5 (http://graphpad-prism.software.informer.com/5.01), we calculated the equilibrium dissociation constant (K_D).

For more details of our protocol for *in vitro* measurements of the equilibrium dissociation constant for TBP–ODN complexes, one can see Ref. [41].

References

34. Ponomarenko, M., Ponomarenko, J., Frolov, A., Podkolodny, N., Savinkova, L., Kolchanov, N., Overton, G. Identification of sequence-dependent features correlating to activity of DNA sites interacting with proteins. *Bioinformatics*. **1999**; 15, 687-703.
36. Ponomarenko, P., Savinkova, L., Drachkova, I., Lysova, M., Arshinova, T., Ponomarenko, M., Kolchanov, N. A step-by-step model of TBP/TATA box binding allows predicting human hereditary diseases by single nucleotide polymorphism. *Dokl. Biochem. Biophys*. **2008**; 419, 88-92.
40. Savinkova, L., Drachkova, I., Arshinova, T., Ponomarenko, P., Ponomarenko, M., Kolchanov, N. An experimental verification of the predicted effects of promoter TATA-box polymorphisms associated with human diseases on interactions between the TATA boxes and TATA-binding protein. *PLoS One.* **2013**; 8, e54626.
41. Drachkova, I., Savinkova, L., Arshinova, T., Ponomarenko, M., Peltek, S., Kolchanov, N. The mechanism by which TATA-box polymorphisms associated with human hereditary diseases influence interactions with the TATA-binding protein. *Hum. Mutat*. **2014**; 35, 601-608.
45. Ponomarenko, M., Rasskazov, D., Arkova, O., Ponomarenko, P., Suslov, V., Savinkova, L., Kolchanov, N. How to use SNP_TATA_Comparator to find a significant change in gene expression caused by the regulatory SNP of this gene's promoter via a change in affinity of the TATA-binding protein for this promoter. *Biomed. Res. Int*. **2015**; 2015, 359835.
181. Hahn, S., Buratowski, S., Sharp, P., Guarente, L. Yeast TATA-binding protein TFIID binds to TATA elements with both consensus and nonconsensus DNA sequences. *Proc. Natl. Acad. Sci. USA*. **1989**; 86, 5718–5722.
182. Bucher, P. Weight matrix descriptions of four eukaryotic RNA polymerase II promoter elements derived from 502 unrelated promoter sequences. J, Mol, Biol. 1990; 212, 563-578.
183. Karas, H., Knuppel, R., Schulz, W., Sklenar, H., Wingender, E. Combining structural analysis of DNA with search routines for the detection of transcription regulatory elements. *Comput. Applic. Biosci*. **1996**; 12, 441-446.
184. Pugh, B. Purification of the human TATA-binding protein, TBP. *Methods Mol. Biol*. **1995**; 37, 359–367.

© 2019 by the authors. Submitted for possible open access publication under the terms and conditions of the Creative Commons Attribution (CC BY) license (http://creativecommons.org/licenses/by/4.0/).
